# FGF8–FGFR1 signaling regulates human GnRH neuron differentiation in a time- and dose-dependent manner

**DOI:** 10.1242/dmm.049436

**Published:** 2022-08-16

**Authors:** Venkatram Yellapragada, Nazli Eskici, Yafei Wang, Shrinidhi Madhusudan, Kirsi Vaaralahti, Timo Tuuri, Taneli Raivio

**Affiliations:** 1 Stem Cells and Metabolism Research Program (STEMM), Research Programs Unit, Faculty of Medicine, University of Helsinki, Biomedicum 1, 00290 Helsinki, Finland; 2 Medicum, Department of Physiology, Faculty of Medicine, University of Helsinki, Haartmaninkatu 8, 00290 Helsinki, Finland; 3 Department of Obstetrics and Gynecology, 00290 Helsinki University Hospital, Helsinki, Finland; 4 New Children's Hospital, Pediatric Research Center, Helsinki University Central Hospital, Stenbäckinkatu 9 Rakennus 6, 00290 Helsinki, Finland

**Keywords:** hPSCs, GnRH neuron, FGF8, FGFR1, Transcriptome

## Abstract

Fibroblast growth factor 8 (FGF8), acting through the fibroblast growth factor receptor 1 (FGFR1), has an important role in the development of gonadotropin-releasing hormone-expressing neurons (GnRH neurons). We hypothesized that FGF8 regulates differentiation of human GnRH neurons in a time- and dose-dependent manner via FGFR1. To investigate this further, human pluripotent stem cells were differentiated during 10 days of dual-SMAD inhibition into neural progenitor cells, followed either by treatment with FGF8 at different concentrations (25 ng/ml, 50 ng/ml or 100 ng/ml) for 10 days or by treatment with 100 ng/ml FGF8 for different durations (2, 4, 6 or 10 days); cells were then matured through DAPT-induced inhibition of Notch signaling for 5 days into GnRH neurons. FGF8 induced expression of *GNRH1* in a dose-dependent fashion and the duration of FGF8 exposure correlated positively with gene expression of *GNRH1* (*P*<0.05, *Rs*=0.49). However, cells treated with 100 ng/ml FGF8 for 2 days induced the expression of genes, such as *FOXG1*, *ETV5* and *SPRY2*, and continued FGF8 treatment induced the dynamic expression of several other genes. Moreover, during exposure to FGF8, FGFR1 localized to the cell surface and its specific inhibition with the FGFR1 inhibitor PD166866 reduced expression of *GNRH1* (*P*<0.05). In neurons, FGFR1 also localized to the nucleus. Our results suggest that dose- and time-dependent FGF8 signaling via FGFR1 is indispensable for human GnRH neuron ontogeny.

This article has an associated First Person interview with the first author of the paper.

## Abstract (precis)

**Summary:** This article demonstrates the essential role FGF8–FGFR1 signaling has in the development of gonadotropin-releasing hormone (GnRH)-expressing neurons by using a human stem cell model.

## INTRODUCTION

Fibroblast growth factor (FGF) signaling via the FGF receptor (FGFR) family of tyrosine kinases is an essential component of mammalian growth and development (Brewer et al., 2016). FGF signaling precedes the activation of signaling pathways including those of MAPK/ERK, PI3K–AKT and PLCγ, which regulate processes, such as cell survival, proliferation, migration and differentiation (Mossahebi-Mohammadi et al., 2020). The FGF family of proteins, which consists of a total of 22 members, is further divided into seven subfamilies. Fibroblast growth factor 8 (FGF8), together with FGF17 and FGF18, belongs to the FGF8 subfamily (Ornitz and Itoh, 2015; Hui et al., 2018). In humans, alternative splicing of *FGF8* results in four isoforms, i.e. *FGF8a*, *FGF8b*, *FGF8e* and *FGF8h* (Sunmonu et al., 2011) and, in terms of brain patterning, *FGF8a* and *FGF8b* have been studied most (Olsen et al., 2006; Sunmonu et al., 2011). Of these two, *Fgf8b* is the predominantly expressed isoform of FGF8 (Sunmonu et al., 2011; Westphal et al., 2019), also holding a stronger signaling activity via its cognate FGFRs (Olsen et al., 2006; Falardeau et al., 2008).

Hypothalamic gonadotropin-releasing hormone (GnRH)-expressing neurons, i.e. GnRH neurons, play an instrumental role in coordinating the activity of the hypothalamic–pituitary–gonadal (HPG) axis and maintaining the reproductive cycle in mammals. GnRH activates the pituitary gonadotrophs to produce luteinizing hormone and follicle-stimulating hormone (Kaprara and Huhtaniemi, 2018). Interestingly, GnRH neurons develop outside the central nervous system and first emerge in the olfactory-placode and olfactory-pit area, from where they migrate to the hypothalamus (Wray et al., 1989; Cho et al., 2019). Animal studies have shown that *Fgf8* expression in the olfactory placode-related neurogenic niche is essential for GnRH neurogenesis and formation of GnRH neurons (Chung et al., 2008; Chung and Tsai, 2010; Forni et al., 2013; Chung et al., 2016). FGF8 is a crucial morphogen in early brain development – especially in the patterning of the anterior telencephalon (Sato et al., 2017), including olfactory bulb development (Hébert et al., 2003) – and its deficiency causes defects in olfactory neurogenesis (Kawauchi et al., 2005). Loss of *FGF8* causes delayed/absent puberty (Chung et al., 2008; Falardeau et al., 2008), which is attributable to the lack of GnRH neurons (Chung et al., 2008; Falardeau et al., 2008). During formation of the olfactory bulb and GnRH neurons, FGF8 acts mainly via FGFR1, i.e. one of the four FGF receptors (Mott et al., 2010; Tsai et al., 2011; Linscott and Chung, 2019) and its three isoforms (FGFR1-IIIa, FGFR1-IIIb and FGFR1-IIIc). Isoform FGFR1-IIIa encodes a receptor without signaling activity, whereas FGFR1-IIIb and FGFR1-IIIc act in a tissue-specific manner with a selective preference towards FGF ligands (Zhang et al., 2006). *Fgfr1*-knockout mice are embryonic lethal (Deng et al., 1994; Xu et al., 1999) and selective telencephalic ablation of *Fgfr1* causes agenesis of the olfactory bulb (Hébert et al., 2003). In addition, *FGFR1* has been implicated in the migration and function of GnRH neurons (Hu et al., 2013). In humans, mutations in *FGF8* and *FGFR1* are known to cause congenital hypogonadotropic hypogonadism (CHH) without or with anosmia, i.e. CHH+anosmia (Young et al., 2019), septo-optic dysplasia or combined pituitary hormone deficiency (Raivio et al., 2012), Hartsfield syndrome (Palumbo et al., 2019), holoprosencephaly and split hand/foot malformation (Villanueva et al., 2015).

Human GnRH neurons have remained largely inaccessible, which has hindered the research to understand their origin and specification (Casoni et al., 2016; Rigobello and Roberto, 2017). Human pluripotent stem cells (hPSCs) have unlimited self-renewal and differentiation capacity (Takahashi et al., 2007; Hay et al., 2008). We have previously described a protocol to differentiate hPSCs into GnRH neurons by specification of neuronal precursors through concomitant inhibition of the BMP and TGFβ pathways, i.e. dual-SMAD inhibition (Chambers et al. 2009), followed by treatment with FGF8 to induce GnRH neurons and inhibition of Notch signaling (Lund et al., 2016). Subsequently, we described the transcriptome of hPSC-derived GnRH neurons (Lund et al., 2020) and investigated the role of ablation of *MKRN3* has in the development of GnRH neurons by using CRISPR/Cas9 technology (Yellapragada et al., 2019). Given that the dose and temporal effects of FGF8 in our protocol have not been explored, we set out to investigate these on the fate-determination of neuronal precursors while they differentiate into GnRH neurons. Finally, we investigated the transcriptomic changes triggered by FGF8 in order to identify those genes and networks that are activated by FGF8.

## RESULTS

### The relationship between FGF8 exposure and *GNRH1* gene expression

**Fig. 1.**
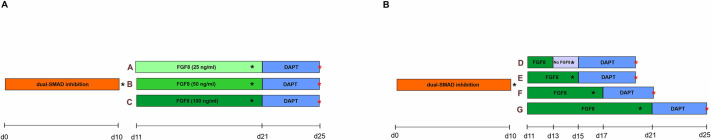
**Schematic of the 25-day GnRH neuron differentiation protocol and dose–time-series experiments.** (A,B) In both experimental set-ups, the differentiation protocol starts with 10 days of dual-SMAD inhibition. In set-up A, this was followed on day 11 by treatment with 25 ng/ml, 50 ng/ml or 100 ng/ml FGF8 for 10 days (conditions A, B or C, respectively). In set-up B, dual-SMAD inhibition was followed on day 11 by treatment with 100 ng/ml FGF8 for different durations, i.e. for 2, 4, 6 or 10 days (conditions D,E, F or G, respectively). After treatment with FGF8, cells were matured by addition of the Notch inhibitor DAPT for 5 days (Lund et al., 2016). Black asterisk*,* cell splitting; red asterisk, end of experiment.

**Fig. 2.**
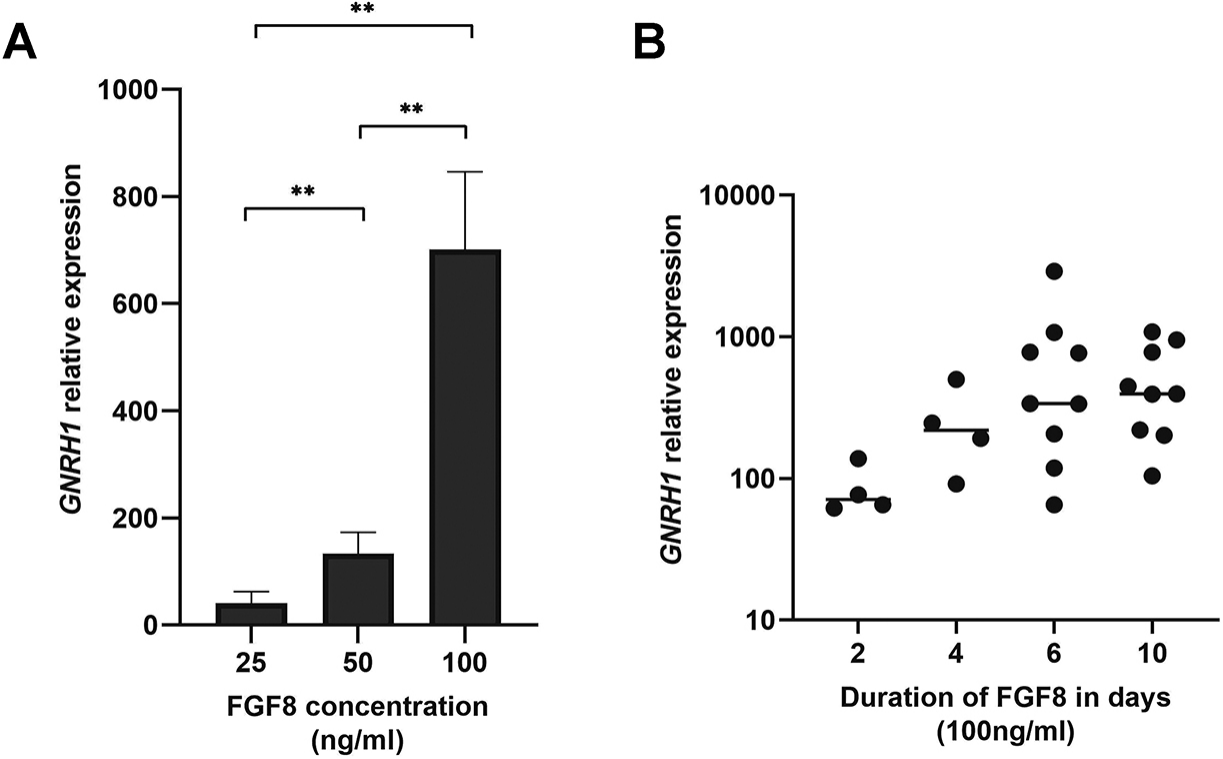
**FGF8 dose and time effect on *GNRH1* expression.** (A) Relative expression of *GNRH1* at day 25 of the differentiation protocol in response to increasing FGF8 concentrations between day 11 and day 21 (see Fig. 1, conditions D-G in Fig. 1). One-way ANOVA indicated a **P≤0.01 between sample sets. Differentiation experiments were repeated three times (*n*=3). Error bars indicate +standard error of the mean (+s.e.m.). (B) Relationship between the duration of treatment with FGF8 (at 100 ng/ml) and the relative expression of *GNRH1* at day 25 of the differentiation experiment (Spearman’s rank correlation coefficient *R_S_*=0.49; *P*=0.01). Each dot represents the relative expression of *GNRH1* from a single experiment (*n*=4, for 2 and 4 days of treatment with FGF8; *n*=9 for 6 and 10 days of treatment with FGF8.). Treatment with 100 ng/ml FGF8 for 2 days induced significant expression of *GNRH1* (*P*<0.05). Horizontal bars indicate median values.

**Fig. 3.**
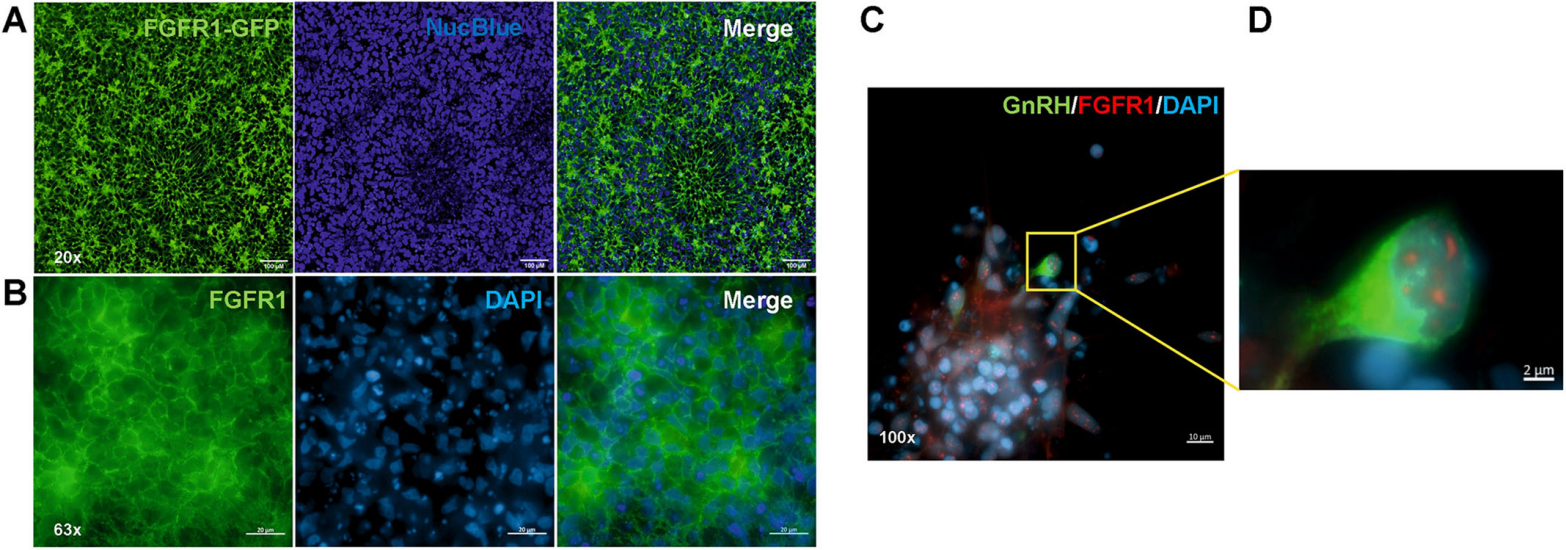
**Cellular expression of FGFR1 during differentiation to GnRH neurons.** (A) Localization of FGFR1 in an *FGFR1*-*GFP* reporter cell line on day 11 of the differentiation protocol. Green, FGFR1-GFP fluorescence; blue, NucBlue™ Live ReadyProbes ™ cell-permeant dye used to counterstain nuclei. Scale bars: 100 μm. (B) Immunostaining of FGFR1 (green) on day 17 (6 days after treatment with FGF8 at 100 ng/ml) of the differentiation experiment, indicating its localization to the cell membrane. Nuclei are shown in blue (DAPI). Scale bars: 20 μm. (C,D) Immunostaining for FGFR1 (red) on day 25 of the differentiation, showing its nuclear localization in neurons, including GnRH neurons (green) is shown C. The boxed area is shown magnified in D, demonstrating the nuclear localization of FGFR1 in a *GNRH1*-expressing neuron. Nuclei are shown in blue (DAPI). Scale bars: 10 μm (C), 2 μm (D).

**Fig. 4.**
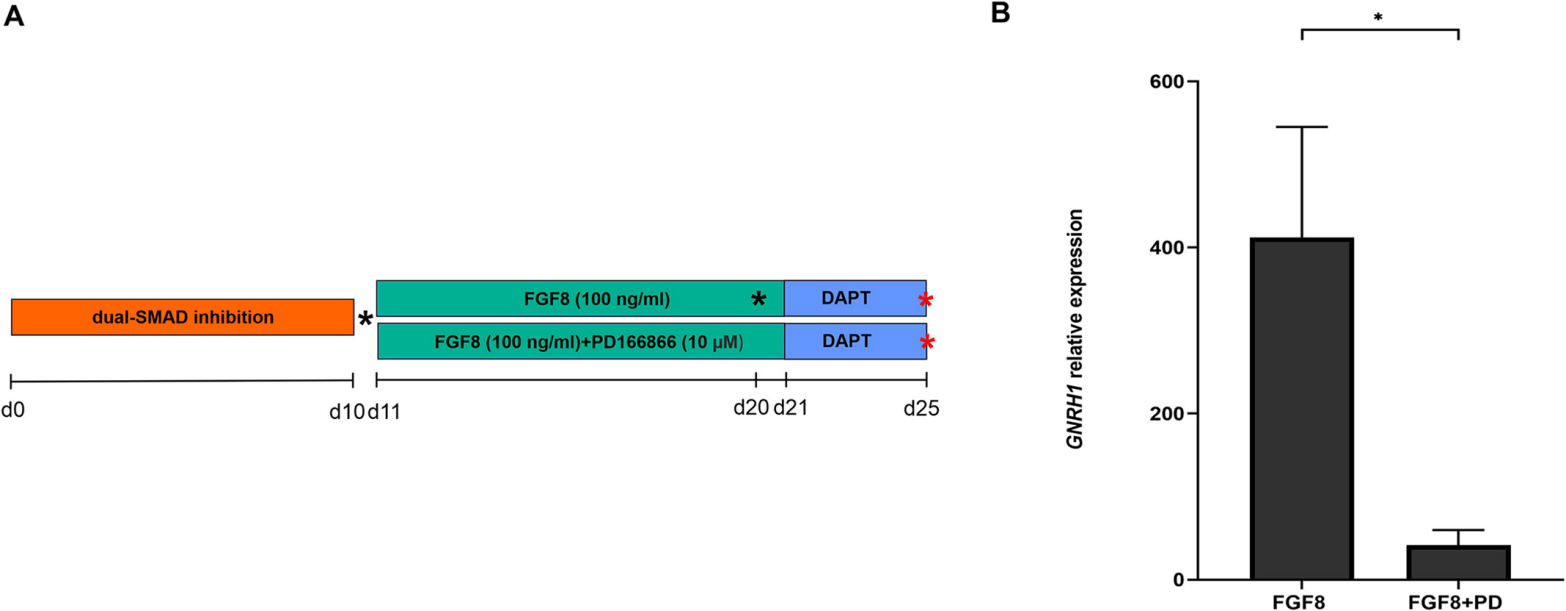
**Effect of FGFR1 inhibition on the relative gene expression of *GNRH1*.** (A) Schematic of the differentiation protocol when cells were treated with 100 ng/ml FGF8 alone or with FGF8 and 10 µM of the FGFR1 inhibitor PD166866 (PD); black asterisk, cell splitting; red asterisk, end of differentiation. (B) Bar graphs show the relative levels of *GNRH1* RNA obtained from cells at day 25 of the differentiation protocol, after treatment with FGF8 alone or FGF8+PD compared to undifferentiated hPS control cells. (*n*=4, *P<0.05; error bars indicate the +standard error of the mean (+s.e.m.).

Neural progenitor cells were treated with FGF8b (referred to as FGF8 hereafter) at three different concentrations, i.e. 25 ng/ml, 50 ng/ml and 100 ng/ml, between days 11 and 21 of the differentiation protocol (Fig. 1A, conditions A-C). A clear dose–response effect (*P*≤0.01) on relative expression of gonadotropin releasing hormone 1 (*GNRH1*) was observed (Fig. 2A)*.* We next investigated the relationship between duration of treatment with FGF8 (100 ng/ml FGF8 for 2, 4, 6 and 10 days, between days 11 and 21, Fig. 1B, conditions D-G) and relative *GNRH1* expression at the end of the protocol. Even 2 days of treatment with FGF8 significantly induced *GNRH1* expression (*P*<0.05) compared to our control; additionally, there was a significant positive Spearman's rank correlation coefficient (*R_S_*) between the duration of FGF8 treatment and expression of *GNRH1* at the end of the culture period (*R_S_*=0.49, *P*=0.01) (Fig. 2B). Interestingly, FGFR1, a crucial receptor for FGF8 during GnRH neuron development, localized to the cell surface upon treatment with FGF8 (100 ng/ml) during the differentiation protocol (Fig. 3A,B, Fig. S1a,b) but, in neuronal cells (including GnRH neurons), it also localized to the nucleus (Fig. 3C,D, Fig. S1a,b). Immunocytochemistry and live imaging suggested day 20 of the differentiation experiments to be the earliest time point at which FGFR1 can be seen localizing to the nucleus (Fig. S1a,b). Finally, to evaluate the role of FGFR1 in FGF8 signaling, we treated cells with the specific FGFR1 blocker PD166866 (10 μM), together with FGF8 (100 ng/ml) between days 11 and 21 of the protocol (Fig. 4A). At the end of the differentiation, PD166866 had significantly reduced *GNRH1* expression (*P*<0.05) (Fig. 4B). Inhibition of FGFR1 accelerated the appearance of cells with bipolar morphology (Fig. S2) but, at the end of the differentiation, both PD-treated and non-treated cells showed neuronal morphology (Fig. S2). Compared with that in control cells, the increase in gene expression of the neuronal marker *MAP2* was similar, i.e. not significant (*P*=ns), in PD166866-treated and untreated cells (Fig. S2).

### Early transcriptomic changes upon treatment with FGF8

**Fig. 5.**
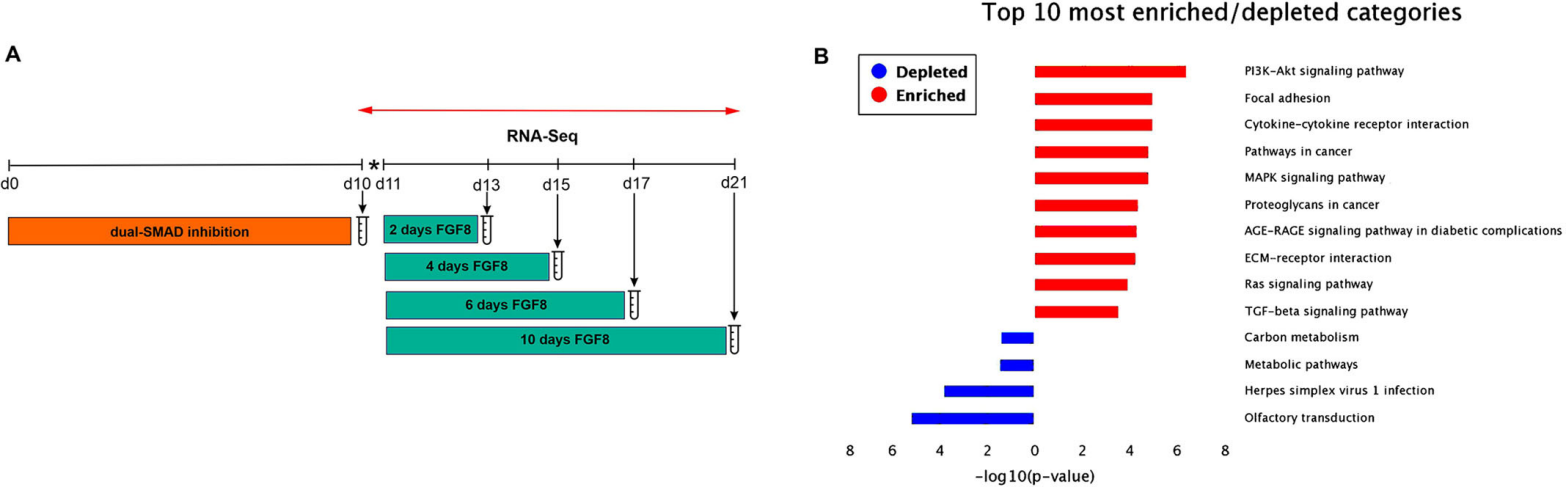
**RNA sequencing strategy and over-representation analysis.** (A) Schematic illustrating the five bulk RNA-Seq time points; dual-SMAD inhibition and FGF8-treated (100 ng/ml) samples after 2, 4, 6 and 10 days, starting at day 11 of the differentiation protocol. Asterisk indicates cell splitting; red double arrow indicates all the RNA-Seq sample timepoints. (B) Results of over-representation analysis (ORA) of upregulated genes after 2 days of FGF8 (d13), represented as a bar chart showing the most-enriched KEGG pathways (red) and the most-depleted ones (blue) on the *y*-axis, and their respective *P*-values on the *x*-axis.

**Fig. 6.**
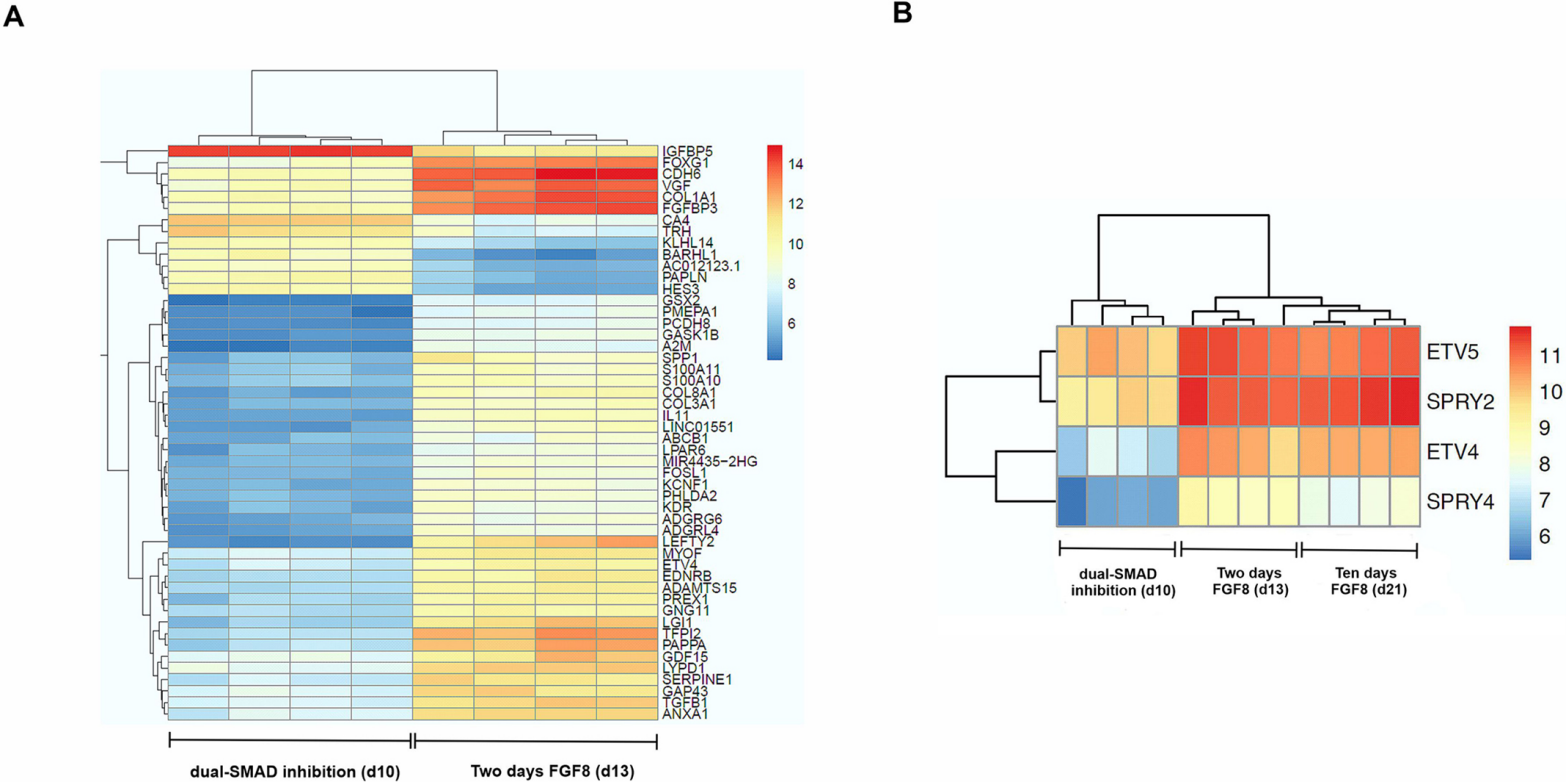
**Early transcriptomic changes after 2 days of FGF8.** (A) Heatmap of the top 50 differentially expressed genes after 2 days of treatment with 100 ng/ml FGF8 (d13) in comparison with dual-SMAD inhibition (d10). (B) Heatmap, showing the differential expression of FGF8 synexpression group genes upon treatment with FGF8. In contrast to their relatively low expression after dual-SMAD inhibition (d10), all four genes were substantially upregulated after treatment with 100 ng/ml FGF8 for 2 or 10 days (d13 or d21, respectively). Colour range: blue (low expression) to red (high expression), numbers indicate the expression scale.

To characterize the transcriptomic changes in FGF8-treated cells, we sequenced RNA that had been extracted at four consecutive time points (Fig. 5A) and performed differential gene expression analyses by using the end of the dual-SMAD inhibition at day 10 (d10) as the reference time point. Given that only 2 days of treatment with FGF8 was sufficient to induce *GNRH1* expression, we first focused on early transcriptomic changes following dual-SMAD inhibition. Two days of treatment with FGF8 (100 ng/ml) resulted in a total of 2804 differentially expressed genes (DEGs) under the set cut-off (*P*≤0.05, absolute log-fold change >1) at which 1937 genes were upregulated and 867 downregulated. Over-representation analysis (ORA) for upregulated DEGs after 2 days of FGF8 treatment showed the most-enriched Kyoto Encyclopedia of Genes and Genomes (KEGG) pathways, i.e. cancer-related pathways, PI3K–Akt signaling, MAPK signaling and TNF signaling, as well as the most-depleted KEGG pathways, i.e. metabolic pathways, herpes simplex virus 1 (HSV1) infection and olfactory transduction (Fig. 5B). Of the affected genes, we found 18 to be direct FGF8 downstream targets, as identified by ingenuity pathway analysis (IPA) (Table S3). The top 50 DEGs included upregulation of genes associated with neuronal differentiation, such as *FOXG1* and *GSX2* (Pei et al., 2011; López-Juárez et al., 2013; Hettige and Ernst, 2019), and downregulation of a neuronal transrepressor *HES3* (Aujla et al., 2011; Hong and Saint-Jeannet, 2018) (Fig. 6A). In addition, FGF8 induced the expression of FGF8 synexpression group genes − i.e. genes for which the protein products are expressed in the same pattern as FGF8 and involved in the same signaling pathways – such as *ETV4*, *ETV5*, *SPRY2* and *SPRY5* (Fig. 6B) (Niehrs and Meinhardt, 2002; Scholpp et al., 2004)*.* When we examined the expression of 32 genes linked to CHH+anosmia (Cho et al., 2019; Messina et al., 2020; Iivonen et al., 2021) after 2 days of FGF8, we found that ten of these genes to be differentially expressed (Fig. S3).

### Differential gene expression during FGF8 treatment

We next investigated DEGs across three other time points of treatment with FGF8 (100 ng/ml), i.e. at d15, d17 and d21. At d15 a total of 3560 DEGs were present (2206 upregulated and 1354 downregulated), at d17 we found a total of 4048 DEGs (2642 upregulated and 1406 downregulated) and at d21 a total of 4009 DEGs (2525 upregulated and 1484 downregulated). Expression of the pro-neuronal genes *FOXG1* and *GSX2* consistently increased, whereas expression of the neuronal transrepressor *HES3* consistently decreased. The expression of FGF8 synexpression group genes stayed either the same or decreased during treatment with FGF8 (Fig. 6B). Next, we examined how many of the DEGs – compared with those after dual-SMAD inhibition – are shared across all four FGF8 treatment time points and found 1595 genes (Table S4). Of those, ∼70% (1109) were upregulated and only 30% (486) were downregulated. We next performed ORA of all upregulated genes across all four time points and found pathways related to cancer, PI3K-Akt signaling and MAPK signaling within the top ten entries to be enriched, whereas HSV1 infection and olfactory transduction pathways were depleted (Fig. S4), which was similar to the results shown after 2 days of FGF8 exposure. We then validated mRNA expression levels of several of the top 50 differentially expressed genes (i.e. *LEFTY2*, *GAP43*, *FOXG1*, *BARHL1* and *HES3*) and of FGF8 synexpression group genes (i.e. *SPRY2* and *ETV5*), together with *ASCL1* and *DLX5* by using qPCR (Fig. S5). Immunostaining on day 17, confirmed expression of two DEGs – *SPRY2* and *NRP1* – shared across all four FGF8 treatment time points*,* both showing the highest upregulation after 6 days of FGF8 (d17) (Fig. S6a). Additionally, immunocytochemistry revealed upregulation of GSX2, ASCL1, FOXG1, LHX2, DLX5 and DCX at protein levels after 2, 6 and 10 days of treatment with 100 ng/ml FGF8 (Fig. S6b,c).

### Dynamic gene expression pathways during treatment with FGF8

In the next step, we investigated whether the DEGs, after 2 days of FGF8 treatment (d13), showed constant or dynamic gene expression pathways, i.e. changing at every time point of RNA-sequencing (RNA-Seq), during treatment with FGF8. We identified a total of 84 DEGs with different dynamic gene expression pathways, the probability of a particular pathway being >95% (Table S5). We focused on the pathways Up-Down-Down-Down, Up-Up-Down-Down, Up-Up-Up-Down and Up-Up-Up-Up (no genes in this category under the particular pathway cut-off >0.95), which we considered to exhibit dynamic upregulation by FGF8. By focusing on these four pathways, we identified a total of 24 genes (Table S6). The dynamic expression pathways of *MCHR1*, *PCDH8*, *SOX14*, *DCX*, *LINC00461* and *SLC32A1* are shown in Fig. S7. The mRNA expression of genes with dynamic expression pathways described above, i.e. *TOX*, *DCX*, *ABAT, TH* and *PCDH8*, was validated by using qPCR and is shown in Fig. S7.

### The FGF8–FGFR1 mechanistic network in the transcriptome data

**Fig. 7.**
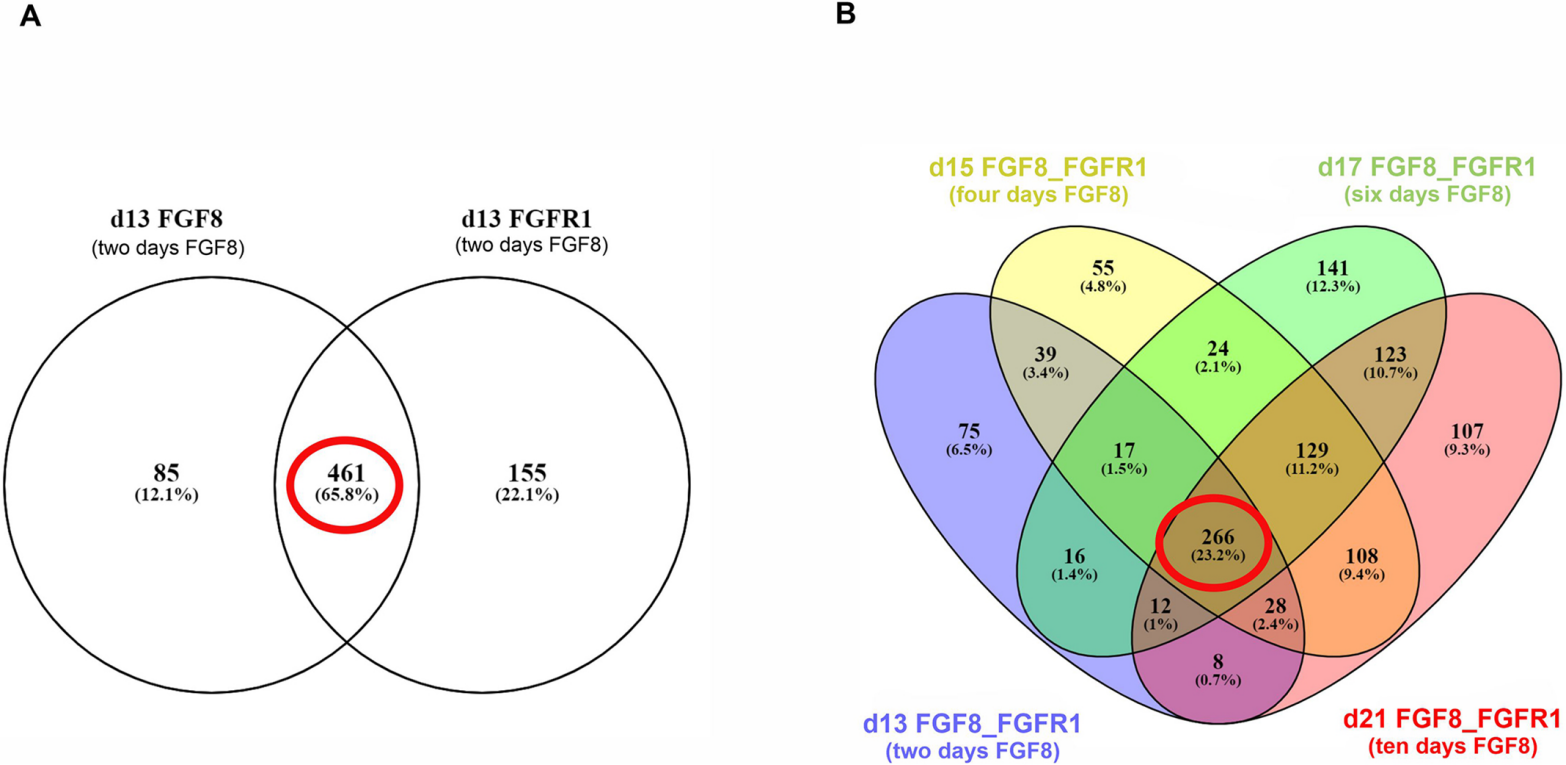
**Analysis of the FGF8–FGFR1 mechanistic network.** (A) Venn diagram indicating the 461 differentially expressed genes (DEGs; encircled in red) on day 13 that belong to FGF8–FGFR1 mechanistic networks as identified by IPA. (B) Venn diagram indicating the DEGs (red circle) across all FGF8 treatment time points that belong to FGF8–FGFR1 mechanistic networks as identified by IPA.

Finally, by using the list of DEGs at day 13 (i.e. after 2 days of FGF8), we performed upstream analysis using IPA and identified possible DEGs in the mechanistic networks of FGF8 and FGFR1, including direct and indirect targets. A total of 546 genes was under the mechanistic network of FGF8, and a total of 616 under that of FGFR1 (Fig. 7A). We next identified overlapping genes within these two networks and found 461 genes, which we anticipated to represent the shared mechanistic network of FGF8 and FGFR1 (Fig. 7A). By repeating the above analysis steps for the other RNA-Seq time points (d15, d17 and d21), we identified three lists of shared FGF8–FGFR1 mechanistic network genes, totaling 666, 728 and 782 genes, respectively. Going further, we identified 266 genes (Fig. 7B) (Table S7) in the shared FGF8–FGFR1 mechanistic network that overlapped across all four time points. As expected, all these 266 genes were among the 1595 shared DEGs, shown in Table S4.

When performing ORA of all the 216 upregulated genes of the 266 DEGs, we observed enrichment in KEGG pathways, such as cancer-related, TNF signaling, MAPK signaling, p53 signaling and PI3K-Akt signaling pathways (Fig. S8), within the top ten; this was coincident with our other ORA results (Fig. 5B, Fig. S4). Additional developmental pathways enriched in this dataset were Hippo signaling and Wnt signaling. Finally, when we compared these 266 genes to the transcriptome of Tdtomato+ human GnRH neurons, 190 of them are, indeed, differentially expressed in GnRH neurons (Lund et al., 2020) with 23 being upregulated and 167 downregulated in GnRH neurons (Table S8).

## DISCUSSION

In this study, we demonstrate a time- and dose-dependent relationship between FGF8 exposure and *GNRH1* expression by using our previously published human pluripotent stem cell-based model for GnRH neuron ontogeny (Lund et al., 2016). The differentiation protocol we employed demonstrated that 100 ng/ml of FGF8 induced the highest *GNRH1* mRNA levels. Interestingly, other hPSC-based GnRH neuron differentiation protocols employed lower FGF8 concentration (10 ng/ml) for a longer duration (Poliandri et al., 2017; Keen et al., 2021) and, contrary to our results, 100 ng/ml was shown to decrease *GNRH1* expression. The precise reason for this discrepancy in FGF8 dosing is currently unclear, but major differences in the differentiation protocols, such as the length of the FGF8 stimulation, may explain the opposing results. In chick embryos, different doses of FGF8 result in different developmental outcomes, particularly in the olfactory and lens placodal precursor cells (Sjödal et al., 2007; Jarrin et al., 2012). In these studies, a low FGF8 dose seems to have no effect on the generation of placodal cells, whereas a higher dose drives the forebrain and neural identity at the expense of placodal cells. Similar studies in mice have indicated that a variation in *Fgf8* levels may affect cell proliferation (Storm et al., 2003) and alter the telencephalic patterning of the developing forebrain (Storm et al., 2006). Other studies have also implicated varying outcomes in the development of the inner ear, forebrain and neocortex upon changes in dose or timing of FGF8 exposure (Fukuchi-Shimogori and Grove, 2001; Storm et al., 2006; Zelarayan et al., 2007; Sato and Joyner, 2009). Sabado et al. explored temporal FGF8 effects on GnRH neuron development in chick embryos (Sabado et al., 2012). Chick GnRH neurons are specified at Hamburger–Hamilton (HH) developmental stage 16, yet the olfactory explants have the potential to already respond to FGF8 at HH15 (Sabado et al., 2012). After GnRH cells are specified at HH17, exposure to FGF8 does not increase the number of GnRH neurons, which suggests a tightly regulated window for the action of FGF8 (Sabado et al., 2012). The human GnRH neuron specification, based on the appearance of immunoreactive GnRH protein, occurs between days 39 and 44 of gestation (Casoni et al., 2016). However, it is currently unknown for how long FGF8 exposure in human embryos is required to prime GnRH precursors *in vivo,* or where *FGF8* is expressed in the developing nasal compartment.

Our results show that even a relatively brief exposure to FGF8 is sufficient to drive neuroepithelial precursor cells to differentiate into human GnRH neurons (Fig. 2B). Specific inhibition of FGFR1 by PD166866 (Yamashita-Sugahara et al., 2016; Yi et al., 2020) reduces *GNRH1* expression drastically (Fig. 4B), indicating an important role for FGFR1 as the primary receptor during human GnRH neuron development. Cells treated with PD166866 seem to acquire bipolar morphology sooner than those treated with FGF8 alone (Fig. S2). This anti-proliferative effect of PD166866 is expected and evident from literature (Risuleo et al., 2009). However, the neural fate of PD166866-treated and untreated cells appeared similar at the end of the differentiation (Fig. S2). During the final stages of FGF8 treatment or, more evidently, after FGF8 treatment when the neurons start to appear in our culture set-up, FGFR1 localized to the nucleus (Fig. S1a,b), suggesting a role in transcription regulation, as previously observed in the development of dopaminergic neurons (Baron et al., 2012). Previously, fluorescence recovery after photobleaching analysis in cell models has demonstrated three separate populations of FGFR1 in the cytoplasm, i.e. those of a highly mobile cytosolic receptor, of a slowly diffusing membrane receptor and of an immobile membrane protein (Dunham-Ems et al., 2006). Additional studies pointed to the likeliness of a highly mobile population of FGFR1 entering the nucleus (Dunham-Ems et al., 2009). Within the nucleus, FGFR1 is associated with an integrative nuclear FGFR1 signaling pathway, especially in the differentiation of neurons from neural progenitor cells (Stachowiak et al., 2003, 2015; Stachowiak and Stachowiak, 2016). Recent studies highlight the role of nuclear FGFR1 in mediating coordinated expression changes for 2851 genes during the differentiation of embryonic stem cells to neuronal committed cells (Decker et al., 2021). Together, these data elevate the possibility of FGFR1 as a transcription factor when localized to the nucleus.

Two days of induction with FGF8 changed the transcriptome of the cells, enriching key developmental pathways, such as TGFβ signaling, PI3K-Akt and MAPK signaling cascades. Of the individual key changes in gene expression, FGF8 synexpression group genes and several genes encoding pro-neuronal transcription factors, such as *GSX2*, *GBX2* and *FOXG1* (Pei et al., 2011; López-Juárez et al., 2013; Wang et al., 2018; Hettige and Ernst, 2019) are upregulated. *Foxg1* is one of the earliest crucial telencephalic transcription factors with its expression induced and/or maintained by FGF8 (Kumamoto and Hanashima, 2017). We have previously shown that migrating human GnRH neurons and hPSC-derived GnRH neurons express FOXG1 (Lund et al., 2016). In mice and zebrafish, *Foxg1* is required for the development of the olfactory system by maintaining progenitors in a proliferative state (Duggan et al., 2008). *Foxg1* is targeted by *miR-9* and *miR200* class microRNAs, which are, in turn, upregulated by *Dlx5* (Garaffo et al., 2015). *DLX5* among several other *DLX* family genes, namely *DLX1*, *DLX2* and *DLX6*, were upregulated after 2 days of treatment with FGF8 in our study. In mice, *Dlx1/2* have been suggested to regulate the number of GnRH neurons (Givens et al., 2005), whereas *Dlx5* has been detected in embryonic GnRH neurons (Givens et al., 2005). Other studies in mice have also shown that *Dlx5* may have a direct role in olfactory neurogenesis (Merlo et al., 2007; Garaffo et al., 2015).

Two days of treatment with FGF8 robustly induced expression of *GSX2*, a transcription factor implicated in forebrain development and olfactory neurogenesis (Roychoudhury et al., 2020; Wen et al., 2021), which regulates the expression of *DLX1*, *DLX2*, *DLX5* and *DLX6* (Pei et al., 2011; Wang et al., 2013; Jain et al., 2001). *GSX2* usually acts upon *GSX1*, *DLX1, DLX2*, *NEUROG1*, *NEUROG2*, *GAD1*, *ASCL1* and *PAX6* (Corbin et al., 2000; Toresson et al., 2000; Jain et al., 2001; Borromeo et al., 2014) and is regulated by *GLI3* (Rallu et al., 2002). Loss of *Gsx2* in mice compromises the expression of *Dlx* family genes and *Ascl1* (Toresson et al., 2000), the latter of which has been implicated in olfactory and GnRH neuron development (Cho et al., 2019; Taroc et al., 2020). *ASCL1* is also upregulated throughout after cells were treated with FGF8 in our current data set. Thus, *GSX2* activation by FGF8 could potentially be a very early and distal factor for GnRH neuron ontogeny. *PAX6* is downregulated upon 2 days of FGF8, with the lowest expression after 10 days of FGF8 (-1.8-fold). *Pax6* is expressed at the nasal-lens domain during early development and its down-regulation is necessary for the specification of the olfactory placode (Bhattacharyya et al., 2004). In addition, FGF8 has been shown to downregulate *Pax6* during early development of chicks (Bertrand et al., 2000). Taken together, even brief exposure of neuronal progenitors to FGF8 induced the expression of transcription factors required for human GnRH neuron ontogeny. Two days of treatment with FGF8 decreased the expression of *HES3,* which together with other HES proteins, acts as an inhibitor of neuronal differentiation and promotes cell proliferation and maintenance of neural stem cell state (Hong and Saint-Jeannet, 2018). Accordingly, inactivation of *Hes1*, *Hes3* and *Hes5* in mouse embryos results in premature neuronal differentiation (Ohtsuka et al., 1999; Hirata et al., 2001). We speculate that the rapid FGF8-induced decline in *HES3* is permissive for the onset of neuronal differentiation.

To date, mutations in more than 30 genes have been implicated in CHH+anosmia, with the underlying mechanisms largely unexplained (Cho et al., 2019). A mechanistic link between these genes would be necessary to understand the development and function of GnRH neurons. Two days of treatment with FGF8 leads to differential expression of ten CHH+anosmia genes *PROKR2*, *FGF8*, *SPRY4*, *FGF17*, *S0X10*, *NRP2*, *SPRY2*, *IL17RD*, *SEMA3A* and *NTN1* (Fig. S3). In our set-up, *NRP1*, a gene implicated in the development of CHH (Men et al., 2021; Oleari et al., 2021), has shown consistent upregulation upon treatment with FGF8. Interestingly, GnRH neurons in mice have been shown to express *Nrp1* (Vanacker et al., 2020), which is known to have a role in the migration of GnRH neurons (Cariboni et al., 2011). In our setup, these changes of gene expression upon treatment with FGF8 highlight the translational value of our stem cell model paradigm in disease modeling.

After 2 days of treatment with FGF8, 84 of the 2804 DEGs exhibited dynamic expression patterns at >95% probability level along the continued FGF8 exposure. Of the 24 genes exhibiting Up-Up-Up-Down, Up-Up-Down-Down or Up-Down-Down-Down patterns was *DCX*, which encodes the neuronal migration protein doublecortin (Fig. S7). Interestingly, DCX protein is also expressed in human fetal GnRH neurons (Casoni et al., 2016). Expression of long intergenic non-protein coding RNA 461 (*LINC00461*) followed the same pathway as expression of *DCX* and exhibited a robust increase in expression after 2 days of treatment with FGF8 (Fig. S7)*. LINC00461* has been implicated in neuropsychiatric disorders and its orthologue in mice regulates neuronal migration (Liu et al., 2020). *SLC32A1* also exhibited an Up-Up-Up-Down gene expression pathway (Fig. S7). It encodes a vesicular inhibitory amino acid transporter (VGAT) that is involved GABA and glycine uptake into the synaptic vesicles, and is highly conserved in the nerve endings of GABAergic neurons (Maejima et al., 2021).

Finally, we investigated which of the genes overexpressed in differentiated GnRH neurons from this protocol (Lund et al., 2020) are upregulated after 2 days of treatment with FGF8 and, thereafter, exhibit dynamic expression patterns. Two transcripts, DLX6 antisense RNA 1 (*DLX6-AS1*) and protocadherin 8 (*PCDH8*), fulfilled these criteria. *DLX6-AS1* represents a long non-coding RNA that has a predictive value in various cancers (Tian et al., 2020). *PCDH8*, in turn, encodes a transmembrane adhesion and signaling molecule that acts as a tumor suppressor (Yu et al., 2020). Intriguingly, *PCDH8* exhibited the highest induction among the DEGs (6.6-fold) after 2 days of FGF8 treatment and, thereafter, exhibited decline, i.e. Up-Down-Down-Down (Fig. S7). *PCDH8* is a target of miR200c (Yu et al., 2020), a member of the miR200 family (see above), and future studies are required to investigate whether miR200 family members modify the *PCDH8* gene expression pathway when our experimental protocol is used. The 266 shared DEGs in the FGF8 and FGFR1 mechanistic networks (Table S7) are a set of unique DEGs, potentially having an important role in GnRH neuron development or function. Among them, *SPRY4*, *GAD1*, *DLX1*, *DLX5* and *DCX* were some of the interesting entries upregulated in FGF8-treated progenitors as well as GnRH neurons. Mutation in *SPRY4* has been shown to cause CHH (Miraoui et al., 2013); and *GAD1,* which converts glutamate into GABA, is also a neurotransmitter controlling GnRH neurons (Di Giorgio et al., 2013).

The DEGs identified here and in a recently published study by Keen et al., (2021) are partly overlapping. For instance, both studies show a decline in the expression of endogenous *FGF8* and *FGFR1* during differentiation, downregulation of *HES3*, and upregulation of several important developmental and differentiation markers, such as *GSX2*, *FOXG1, SOX2*, *ASCL1* and *DLX* family genes, possibly implicating a role of these factors in the differentiation of GnRH neurons. However, as the protocols and sample collection time points are not similar between these two studies, we did not directly compare the sequencing results.

Here, we have shown the crucial role of FGF8–FGFR1 signaling in the differentiation of GnRH neurons from hPSCs together with localization of FGFR1 during and after treatment with FGF8. The differential gene expression data upon FGF8 treatment will help in gaining a better understanding of GnRH neuron development. Finally, the DEGs of the FGF8–FGFR1 mechanistic network identified in our study might serve as a catalog for translational researchers modeling mechanisms of CHH.

## MATERIALS AND METHODS

### Human pluripotent stem cells

H9 human embryonic stem cell (hESC)-based (Thomson et al.,1998) *GNRH1*-Tdtomato reporter cell lines H9C11 and its subclone H9C11D7 (Lund et al., 2020), as well as induced pluripotent stem cell (iPSC) lines HEL11.4 (Mikkola et al., 2012) and HEL24.3 (Trokovic et al., 2015) from healthy donors were used in this study. All hPSCs were maintained on Matrigel-coated dishes (Corning) with mTeSR1 culture medium (STEMCELL Technologies) replaced daily, and kept at 37°C under 5% CO_2_.

### Differentiation of hPSCs into GnRH neurons

At ∼90% confluency, hPSCs were differentiated into GnRH neurons based on our previously published protocol (Lund et al., 2016). N2B27 medium (50% DMEM/F12, Gibco) and 50% Neurobasal medium (Gibco) supplemented with 0.5× N2 (Gibco) and 0.5× B27 (Gibco), 1 mM Glutamax (Gibco) and 1× penicillin-streptomycin, (Sigma) was used as the basal medium throughout the differentiation process. Briefly, for the first 10 days of dual-SMAD inhibition, N2B27 medium was supplemented with 2 µM of the BMP inhibitor Dorsomorphin (Selleckchem) and 10 µM of the TGF-β/activin inhibitor SB431542 (Sigma) to produce neural progenitor cells. From day 11 onwards, culture medium was refreshed daily with N2B27 that had been supplemented with FGF8 (FGF8b, Peprotech) at variable doses (25 ng/ml, 50 ng/ml or 100 ng/ml) and cells were cultured for another 10 days (referred to as conditions A, B and C in Fig. 1A.). Alternatively, for time-series experiments, at day 11 of the differentiation protocol medium was replaced daily with N2B27 containing 100 ng/ml FGF8 and cells were incubated for 2, 4, 6 or 10 days (referred to as conditions D, E, F or G, respectively, in Fig. 1B). At the end of each FGF8 or No FGF8 treatment period, cells were split and treated for another 5 days with the γ-secretase inhibitor 20 µM DAPT (Selleckchem) to indirectly inhibit Notch signaling, with medium being refreshed every second day (see detailed differentiation schematic in Fig. 1).

### Chemical inhibition of FGFR1 activity

The selective FGFR1 inhibitor PD166866 (Selleckchem), was used to block FGFR1 activity (Yamashita-Sugahara et al., 2016; Yi et al., 2020). 10 µM (i.e. 3.96 µg/ml) of PD166866 was used together with 100 ng/ml FGF8 between days 11 and 21 of the differentiation protocol. At day 21, culture medium was replaced with N2B27 containing 20 µM DAPT for 5 days with medium being refreshed every other day (see the illustration of the PD166866-treatment strategy in Fig. 4A).

### RNA isolation, reverse transcription and analysis of *gene* expression

RNA was isolated from the cells collected at multiple time points during differentiation into GnRH neurons by using Nucleospin RNA plus kit (Macherey-Nagel) according to the manufacturer's instructions. 1 µg of total RNA was converted to cDNA using iScript™ cDNA synthesis kit (BIO-RAD) as per the instructions from the manufacturer. By using quantitative PCR, *GNRH1* and *MAP2* RNA levels were first normalized to those of cyclophilin G (*PPIG*) and the relative RNA expression was compared to that in undifferentiated hPSCs. All the primer sequences are provided in Table S1.

### RNA isolation for RNA-Seq samples

For RNA sequencing (RNA-Seq), total RNA was isolated from cells collected at five different time points during the differentiation protocol (Fig. 5A). Briefly, cells were either detached after dual-SMAD inhibition (day 10) or after different durations of treatment with FGF8 (i.e. at days 13, 15, 17 or 21) by using EDTA, and pelleted in ice-cold PBS. RNA was then isolated using the Nucleospin RNA kit (Macherey-Nagel) according to the manufacturer's instructions. RNA was treated with RNase free DNase (Promega) and RNasin Ribonuclease inhibitor (Promega) to deplete genomic DNA.

### Bulk RNA-Seq

RNA was analyzed for concentration, integrity and quality using Qubit Fluorometer (ThermoFisher) and Tapestation 4200 (Agilent). Sequencing was performed as paired end runs for a read length of 150 bp using NovaSeq 6000 sequencer (Illumina) and NEBNext Ultra II Directional PolyA capture library kit (New England BioLabs Inc.). Each sample time point contains four biological replicates and all 20 samples have been sequenced in a single run. RNA quality control and sequencing were performed at the Functional Genomics Unit (FuGu), University of Helsinki, Finland.

### RNA-Seq data processing and analysis

The raw sequencing output in FASTQ format was analyzed for quality by using FASTQC (Simon Andrews, Babraham Bioinformatics, Cambridge, UK) and no additional trimming was performed owing to the good quality of reads after adaptor trimming. Sequencing reads were then aligned against the GENCODE GRCh38 reference (Harrow et al., 2012) using STAR (Dobin et al., 2013). Raw read counts from the aligned bam files were generated using FeatureCounts (Liao et al., 2014) and annotated with Ensembl release 87, using BioMart package in R (Durinck et al., 2009; Yates et al., 2016). From the raw counts, normalization and differential expression analysis were performed using the DESeq2 package in R (Love et al., 2014). We filtered all differentially expressed genes (DEGs), with absolute log-fold changes of >1 and *P*-values <0.05 (Benjamini–Hochberg method). In the list of DEGs, all up- and downregulated genes (based on their log-fold values) were considered separately for over-representation analysis (ORA). ORA was performed with Genetrail 3.2 by using KEGG pathways as biological category and the Benjamini–Hochberg false discovery rate adjustment method (Stöckel et al., 2016).

Ingenuity pathway analysis (IPA, QIAGEN), was employed to perform upstream analysis and to identify DEGs across our dataset in the FGF8–FGFR1 mechanistic networks. For comparative analysis, a previously published human GnRH neuron transcriptome dataset was used (Lund et al., 2020). The dynamic gene expression pathways were identified and visualized using the EBSeqHMM package in R (Leng et al., 2015). EBSeqHMM identified all the DEGs from our data set and plotted their expression across all the five RNA-Seq time points between days 10 and 21. A posterior probability value designated by EBSeqHMM, ranging between 0 and 1, indicates the most probable gene expression path of a DEG: the higher the posterior probability, the higher the probability for a gene to follow that expression path. The change of gene expression in the path of a DEG is calculated with reference to the previous time point where dual-SMAD inhibition (d10) is the first time point. The Venny 2.1 online tool was used for drawing Venn diagrams and to export the data from the Venn diagrams (Carlos, 2015).

### Generation of FGFR1-GFP reporter knock-in to H9 hESCs

The *FGFR1-GFP* reporter cell line was generated using CRISPR-Cas9 genome editing strategy targeting the stop codon of *FGFR1* for insertion of the donor template FGFR1_miniIAA7_mEGFP (Balboa et al., 2017). The donor template included 524 bp (5′) and 501 bp (3′) homologous arms for the introduction of the donor sequence into the genome by homologous recombination. The homologous arms were cloned by PCR from the genomic DNA of the hESC line H9. The insertion fragment was cloned by PCR from the vector (a gift from the laboratory of Elina Ikonen, University of Helsinki, Helsinki, Finland). The fragments were then purified from agarose gel and ligated into a donor template vector by using the NEBuilder HiFi DNA assembly Master Mix (NEB, Cat.E2621S). Guide RNA and Cas9 nucleases were ordered from IDT (Integrated DNA Technologies). Details of the oligonucleotides and plasmids employed are available on request.

### Live cell imaging

After the dual-SMAD inhibition (day 10 of differentiation), the cells were split and plated onto ViewPlate-96 black, optically clear-bottom 96 well plates (PerkinElmer). Next day (day 11), FGF8 (100 ng/ml) was added to the cell culture medium. NucBlue Live ReadyProbes reagent (Invitrogen, ThermoFisher) was added to the culture medium as per the instructions from the manufacturer to detect the nuclear bodies. The cells were imaged at multiple time points during the GnRH neuron differentiation protocol under 20× and 40× water immersion objectives NA 1.0, by using the Opera Phenix spinning disk confocal High-Content Screening System (PerkinElmer) to visualize FGFR1 localization within the cells.

### Immunocytochemistry

Cells from multiple time points during the GnRH neuron differentiation were fixed with 4% PFA on glass coverslips for 15 min at room temperature (RT). The cells were either permeabilized for 7 min in 0.5% Triton X-100 (Sigma-Aldrich) or directly blocked with BlockAid Blocking Solution (Invitrogen, ThermoFisher) to prevent nonspecific binding. After blocking, the cells were incubated with primary antibodies overnight at 4°C and then with secondary antibodies for 1 h at RT. Primary antibodies were: rabbit anti-FGFR1 (Cell Signaling Technology, #9740, 1:200), sheep anti-GnRH1 [a gift from Erik Hrabovszky (Laboratory of Reproductive Neurobiology, Institute of Experimental Medicine, Budapest, Hungary), 1:4000] rabbit anti-GnRH1 (Immunostar, #20075, 1:1000), anti-NRP1 (Abcam, #ab81321, rabbit, 1:250), anti-SPRY2 (Santa Cruz, #sc-100862, mouse, 1:150), anti-GSX2 (Merck, #ABN162, rabbit, 1:500), anti-ASCL1 (Santa Cruz, #sc-28688, rabbit, 1:100), anti-FOXG1 (Abcam, #ab182659, rabbit, 1:1000), anti-LHX2 (Thermo Scientific, #MA5-15834, mouse, 1:200), anti-DLX5 (Abcam, #ab109737, rabbit, 1:500) and anti-DCX (Abcam, #ab18723, rabbit, 1:200). Secondary antibodies were: goat anti-mouse Alexa Fluor 488 (#A32723), donkey anti-mouse Alexa Fluor 488, (#A21202), donkey anti-rabbit Alexa Fluor 488, (#A21206), donkey anti-sheep Alexa Fluor 488 (#A11015), donkey anti-mouse Alexa Fluor 594, (#A21203), donkey anti-rabbit Alexa Fluor 594, (#A21207), donkey anti-mouse Alexa Fluor 594, (#A21203), donkey anti-rabbit Alexa Fluor 594 (#A21207) all from Invitrogen and all used at 1:500). Before mounting, cells were incubated in 0.1 µg/ml DAPI solution (Cat no 62248, ThermoFisher) for 2 min at RT. Coverslips were mounted on microscopic slides with Prolong Diamond Antifade Mountant (Invitrogen, ThermoFisher). Immunostaining images were obtained with a ZEISS Axio Imager.Z2 upright epifluorescence microscope, using 40×, 63× and 100× EC PL APO CS2 oil objectives. All antibodies were diluted in blocking solution and are listed in Table S2.

### Statistics

One-way ANOVA was performed for FGF8 dose–response data. One sample *t*-test and Spearman's correlation test were performed for time-series data. A non-parametric test (Mann–Whitney *U*-test) was performed for PD166866-treatment data. All the statistical tests were performed in GraphPad Prism 8.

## Supplementary Material

Supplementary information
