# Supplementary material for: FGF8–FGFR1 signaling regulates human GnRH neuron differentiation in a time- and dose-dependent manner
Source: Dis Model Mech. 2022 Aug 16;15(8):dmm049436. doi: 10.1242/dmm.049436 (PMC9403748; doi:10.1242/dmm.049436)
Supplement: Supplementary information [file dmm-15-049436-s1.pdf]

Fig. S1a

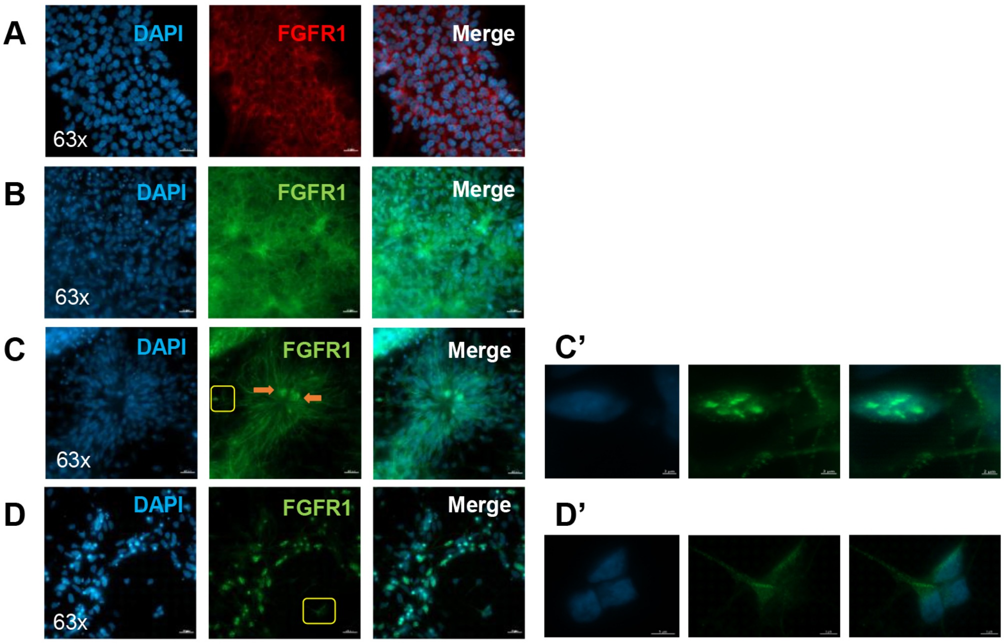

Fig. S1a. FGFR1 immunocytochemistry. Immunostaining of FGFR1 at different time points during the GnRH neuron differentiation protocol starting from day 0 (hPSCs) to day 21 along with nuclear staining by DAPI. Each row represents a time point starting day 0 (A), day 17 (B), day 20 (C) and day 21 before adding DAPT (D). Orange arrows in C indicate cells which have nuclear FGFR1. Panels C' and D' represent an inset of the cells in the yellow boxes of C and D. All images were taken using a 63x objective and the scale bar represents 20 μm (A, B, C, and D), 2 μm (C') and 5 μm (D').

Disease Models & Mechanisms • Supplementary information

Fig. S1b

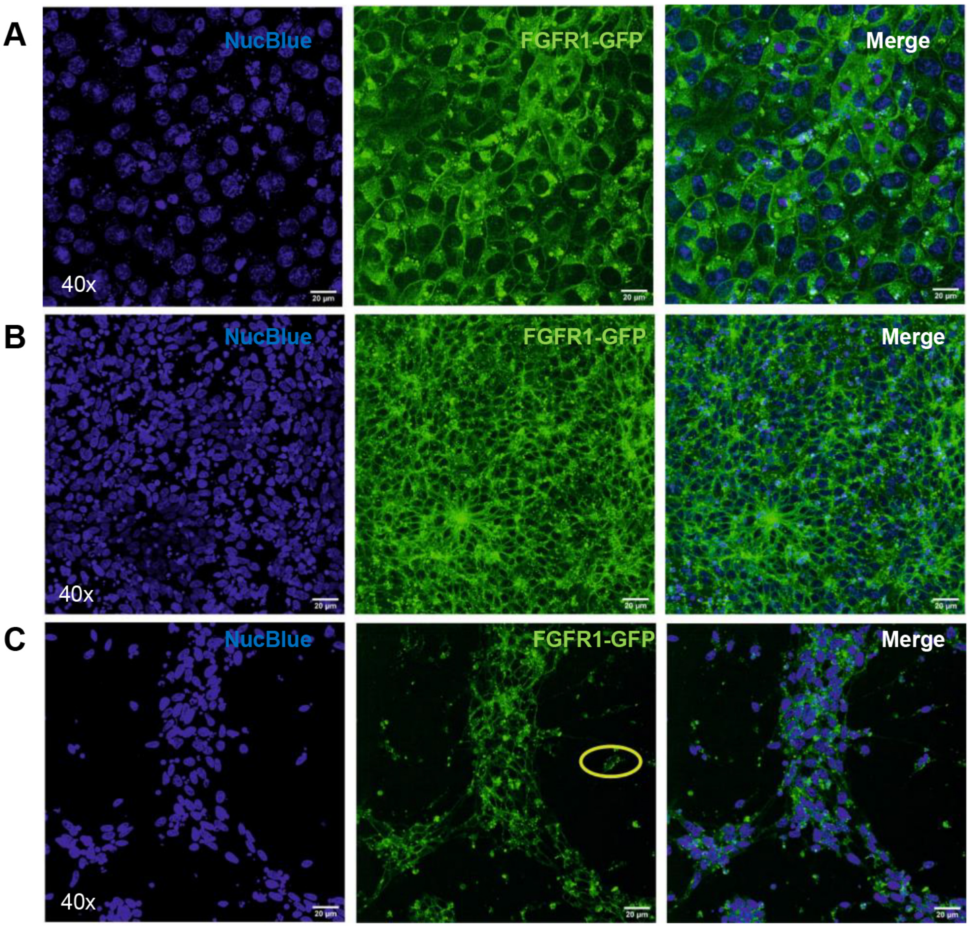

Fig. S1b. Live imaging of FGFR1-GFP reporter cell line. An hPSC based FGFR1-GFP cell line shows FGFR1 localization at day 0 (A), day 20 (B) and day 21 after adding DAPT (C) along with NucBlue™ Live ReadyProbes™ cell-permeant dye to counterstain nuclei. Green: FGFR1-GFP fluorescence; blue: NucBlu. Yellow ellipse in C indicates a cell with FGFR1 nuclear localization. Scale bars represent 20 μm.

Disease Models & Mechanisms • Supplementary information

Fig. S2

A

B

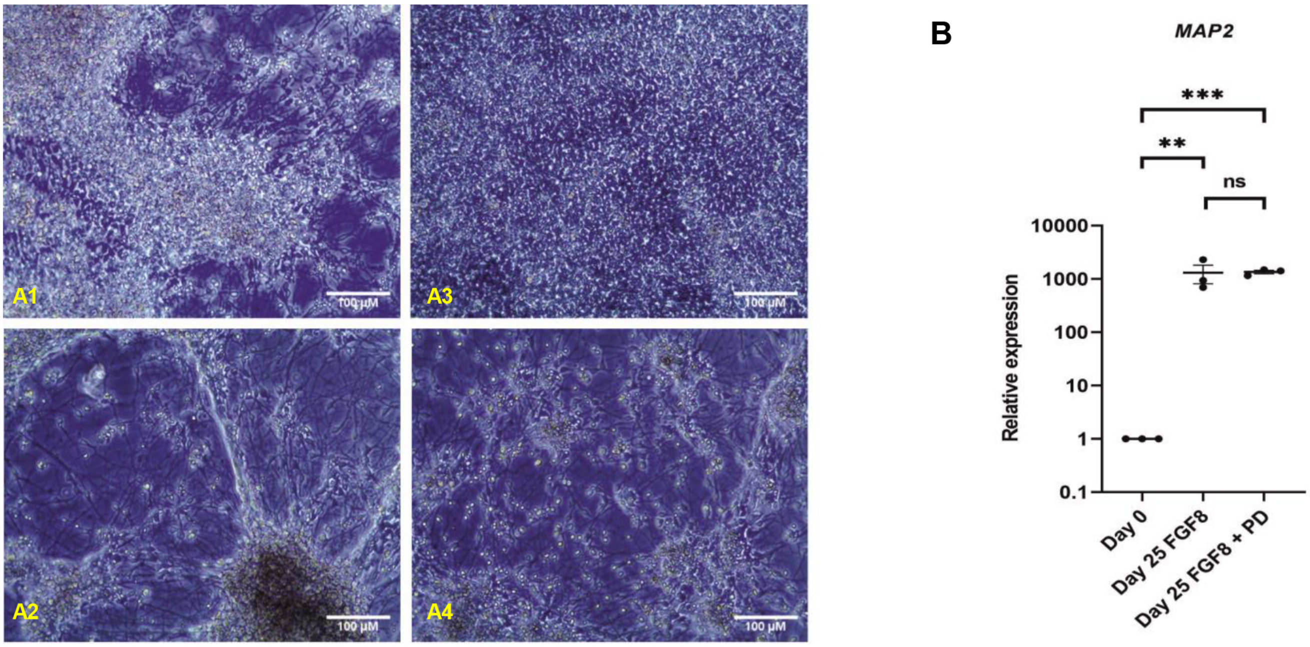

Fig. S2. Morphology and Neural fate of PD166866 (PD) treated cells. (A) Morphology of PD treated (A1 and A2) and untreated (A3 and A4) cells on days 18 and 25 respectively of the GnRH differentiation protocol. (B) Fate of PD treated cells. Day 0, and Day 25 (FGF8 and FGF8 +PD treated) samples were collected from three independent experiments (n=3). MAP2 expression was used an indicative measure for neuronal fate. MAP2 expression levels and cellular morphology of PD treated cells was comparable with FGF8 alone treated cells on day 25. Statistical significance (ratio paired t-test) indicated as *(P≤0.05), **(P≤0.01), ***((P≤0.001), and ns (P>0.05). Related to Fig. 4.

Disease Models & Mechanisms • Supplementary information

Fig. S3

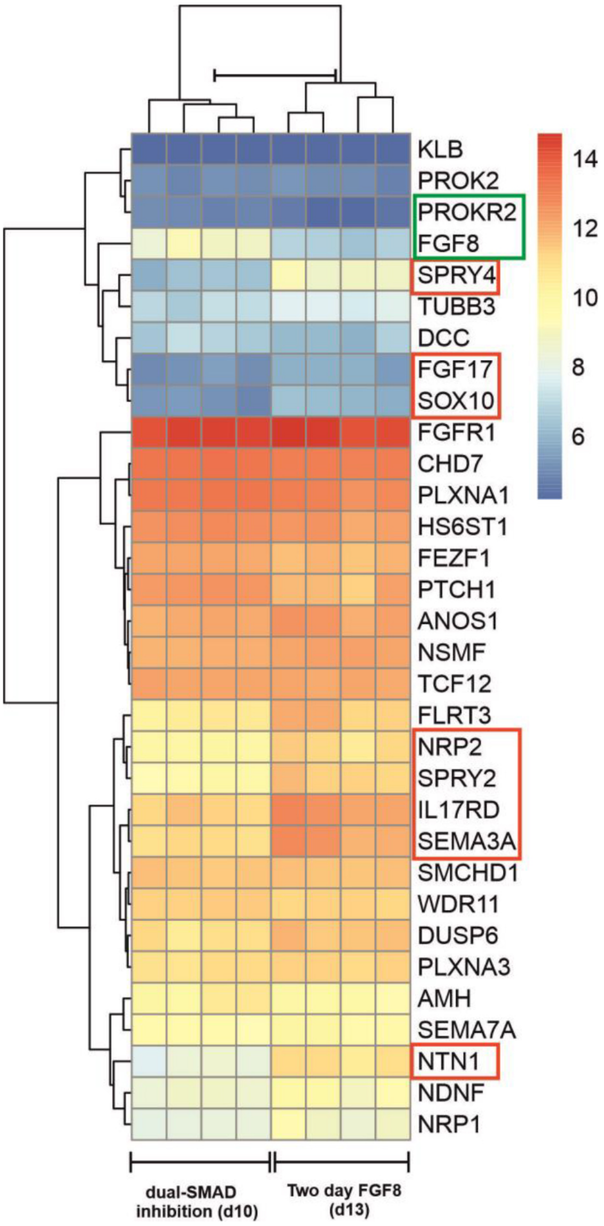

Fig. S3. Expression of CHH + anosmia genes. A heatmap showing the expression of CHH + anosmia genes after two days of FGF8 treatment (d13) compared to dual-SMAD inhibition (d10). Ten among these are differentially expressed, genes in red boxes are upregulated (*SPRY4*, *FGF17*, *SOX10*, *NRP2*, *SPRY2*, *IL17RD*, *SEMA3A*, and *NTN1*) whereas those in green box are downregulated (*PROKR2*, and *FGF8*).

Fig. S4

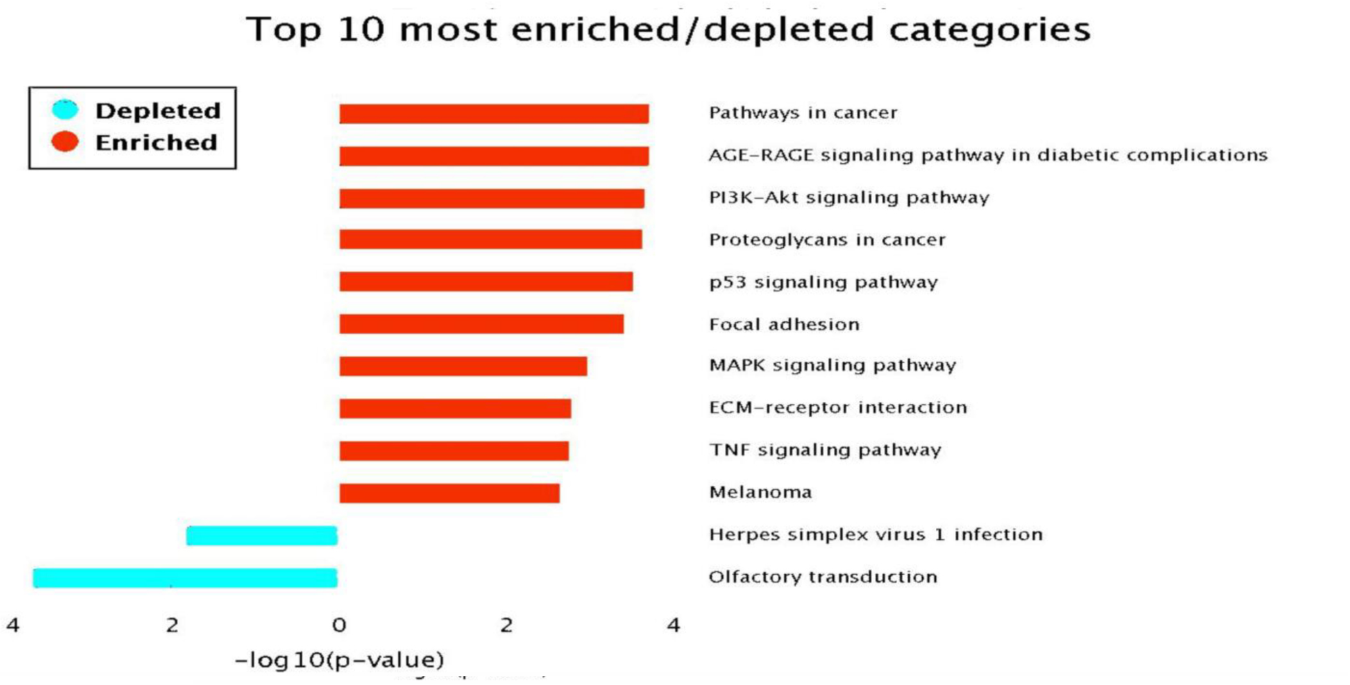

Fig. S4. Over-representation analysis (ORA). All the upregulated DEGs across all four FGF8 treatment time points were subjected to ORA and the most enriched (red) and depleted (blue) KEGG pathways (y-axis), and their respective p-values (x-axis) are presented here. Related to Table S4.

Fig. S5

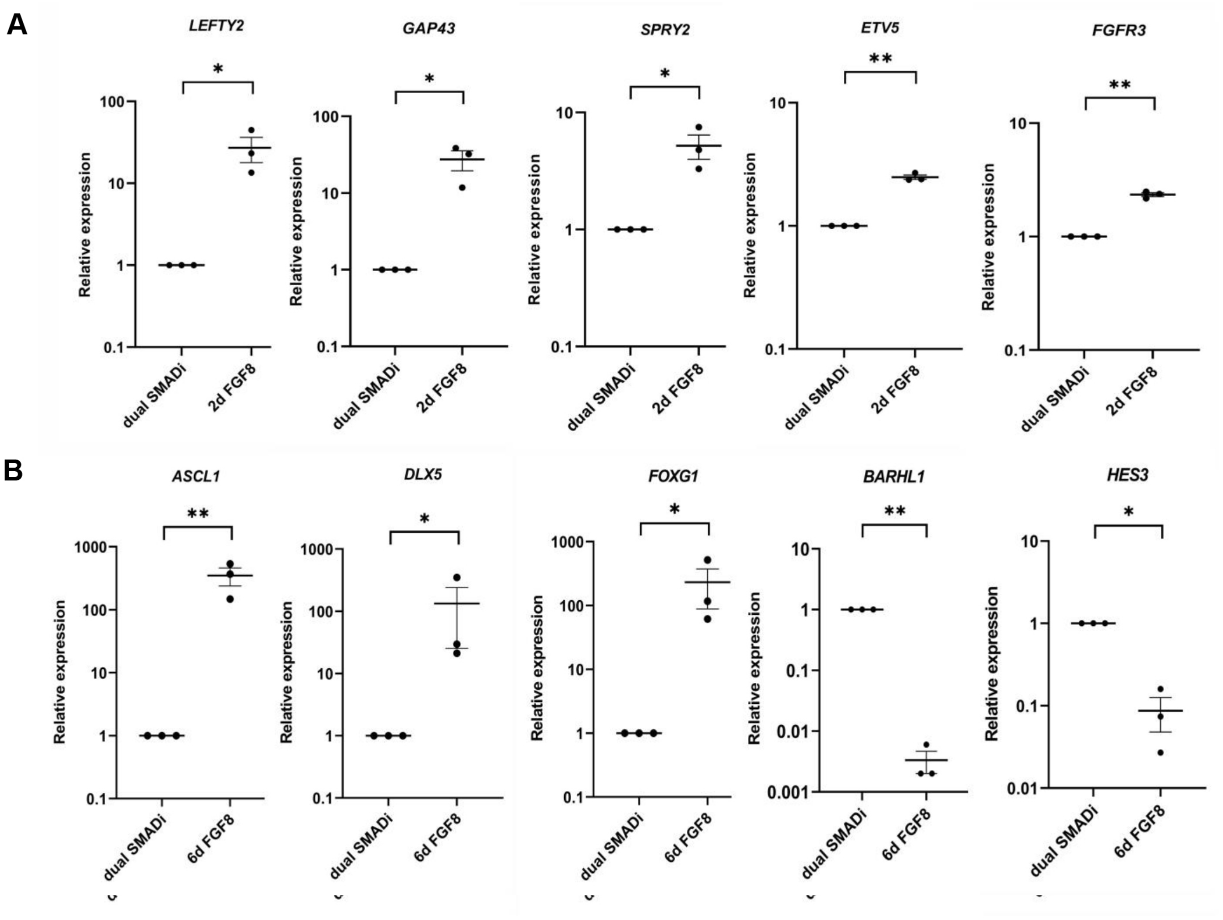

Fig. S5. qPCR validation of differentially expressed genes. (A) *LEFTY2*, *GAP43*, *SPRY2*, *ETV5,* and *FGFR3* were among the upregulated genes after 2d FGF8 (day 13) and they displayed increase in their relative expression levels compared to dual SMADi (dual-SMAD inhibition, day 10). (B) *ASCL1, DLX5, FOXG1* were upregulated whereas, *BARHL1*, and *HES3* were downregulated genes after 6d FGF8 (day 17) and they displayed increase (*ASCL1, DLX5,* and *FOXG1*) and decrease (*BARHL1*, and *HES3*) in their relative expression after 6d FGF8 compared to dual SMADi. Dual SMADi, 2d FGF8 and 6d FGF8 samples were collected from three independent experiments (n=3). Statistical significance (ratio paired t-test) indicated as *(P≤0.05), ** (P≤0.01), and *** (P≤0.001). Related to Fig. 5, 6, and 7 and Table S4.

Disease Models & Mechanisms • Supplementary information

Fig. S6a.

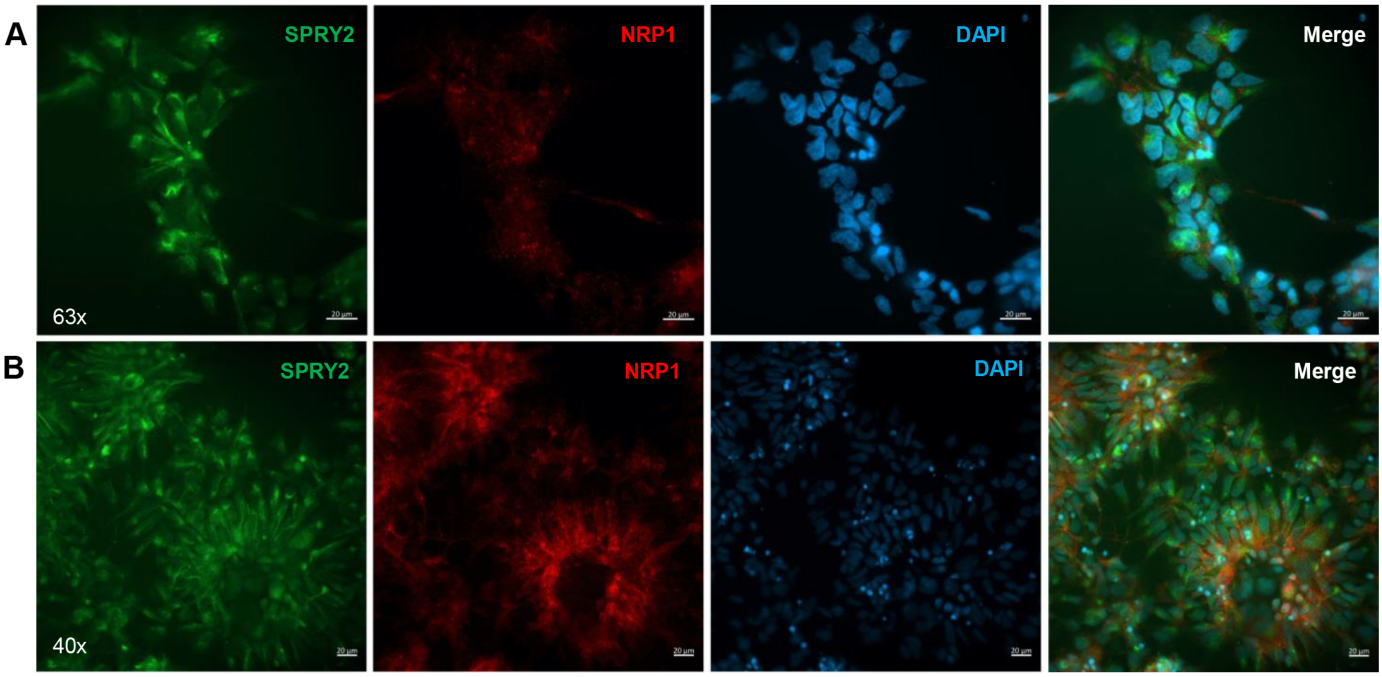

Fig. S6a. Validation of RNA-Seq data with immunocytochemistry. Immunocytochemistry at day 17 (6d FGF8) of anti-SPRY2 and anti-NRP1 along with nuclear staining by DAPI. (A) SPRY2 and NRP1 immunostaining visualized under 63x objective and (B) SPRY2 and NRP1 immunostaining visualized under 40x objective. Scalebars represent 20 μm.

Disease Models & Mechanisms • Supplementary information

Fig. S6b.

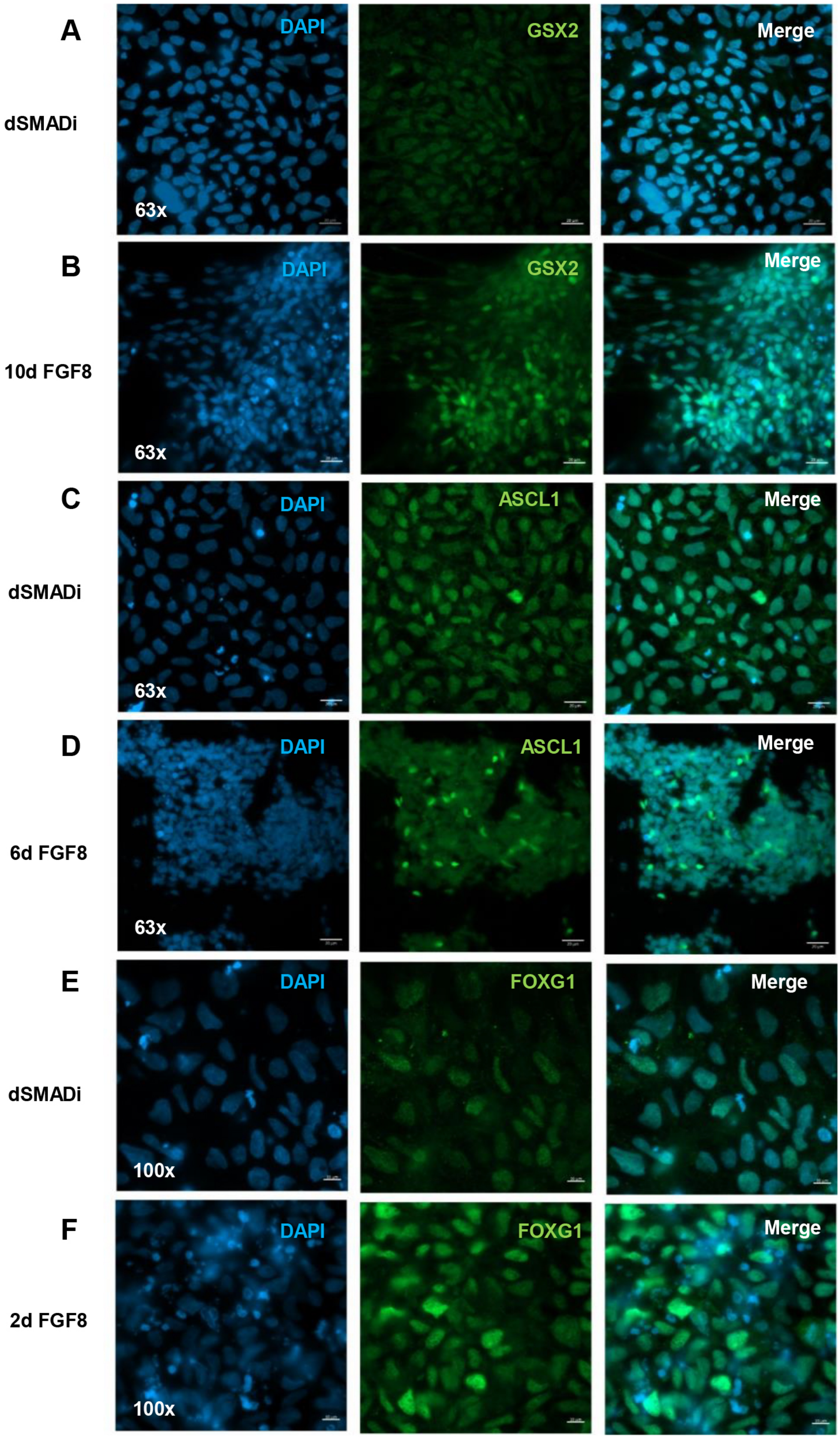
A
dSMADi
DAPI
GSX2
Merge
63x
B
10d FGF8
DAPI
GSX2
Merge
63x
C
dSMADi
DAPI
ASCL1
Merge
63x
D
6d FGF8
DAPI
ASCL1
Merge
63x
E
dSMADi
DAPI
FOXG1
Merge
100x
F
2d FGF8
DAPI
FOXG1
Merge
100x

Fig. S6b. Immunocytochemical validation of differentially expressed genes from RNA-seq data. Immunocytochemistry at days 10 (dual-SMAD ihhibition, dSMADi) and 20 (10d FGF8) of anti-GSX2 (A, B), days 10 and 17 (6d FGF8) of anti-ASCL1 (C, D), and at days 10 and 13 (2d FGF8) of anti-FOXG1 (E, F) along with nuclear staining by DAPI using 63x or 100x objective. Scale bars indicate 20 µm (63x) and 10 µm (100x). Signal intensity for GSX2, ASCL1, and FOXG1 showed increase in line with the RNA-seq data**.**

Fig. S6c.

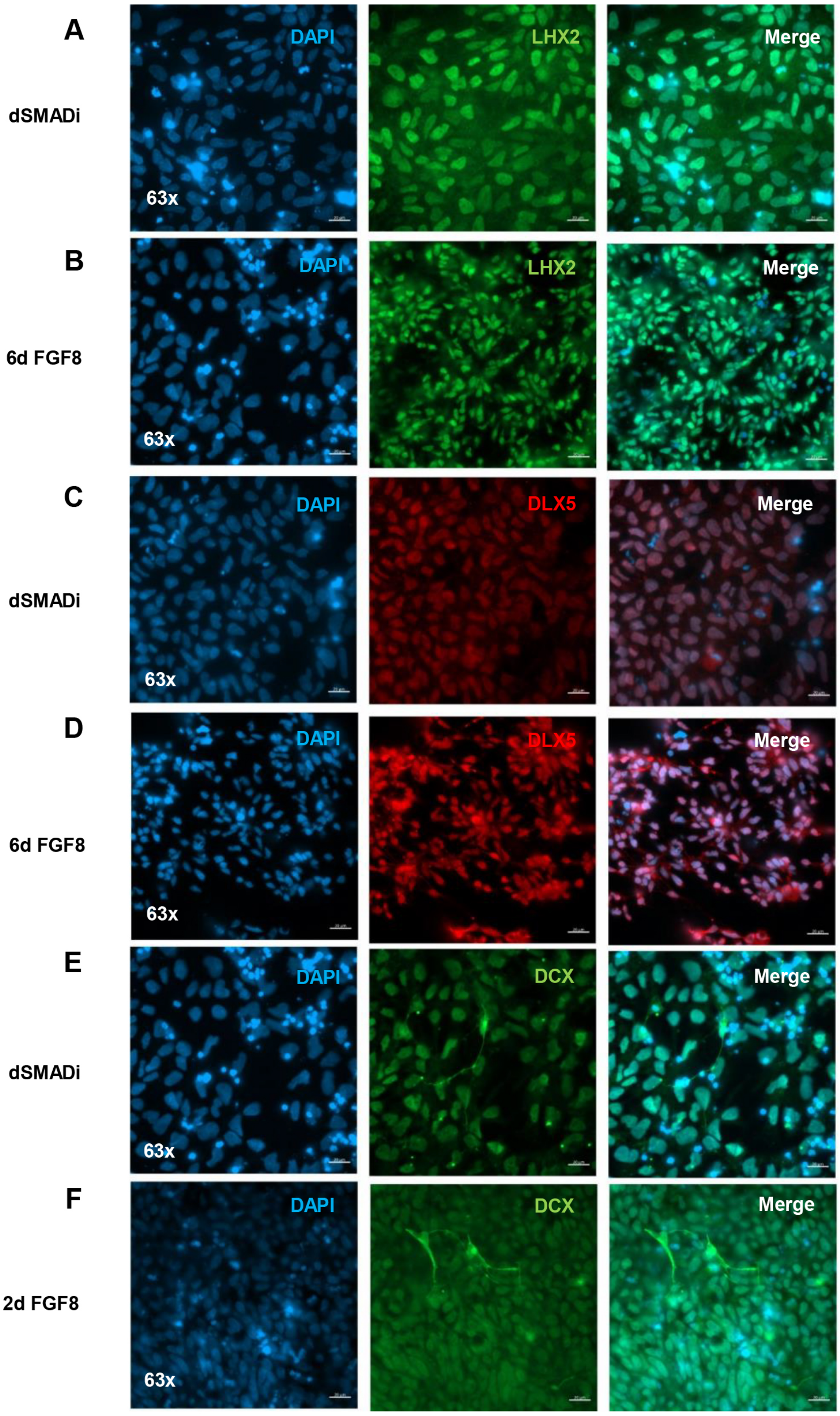

Fig. S6c. Immunocytochemical validation of differentially expressed genes from RNA-seq data. Immunocytochemistry at days 10 (dual-SMAD inhibition, dSMADi) and 17 (6d FGF8) of anti-LHX2 (A, B), anti-DLX5 (C, D), and at days 10 and 13 (2d FGF8) of anti-DCX (E, F) along with nuclear staining by DAPI using 63x objective. Scale bars indicate 20 µm. Signal intensity for LHX2, DLX5, and DCX showed an increase in line with the RNA-seq data. *LHX2* and *DCX* have considerable amount of expression already at day 10 in the RNA-Seq data.

Disease Models & Mechanisms • Supplementary information

Fig. S7.

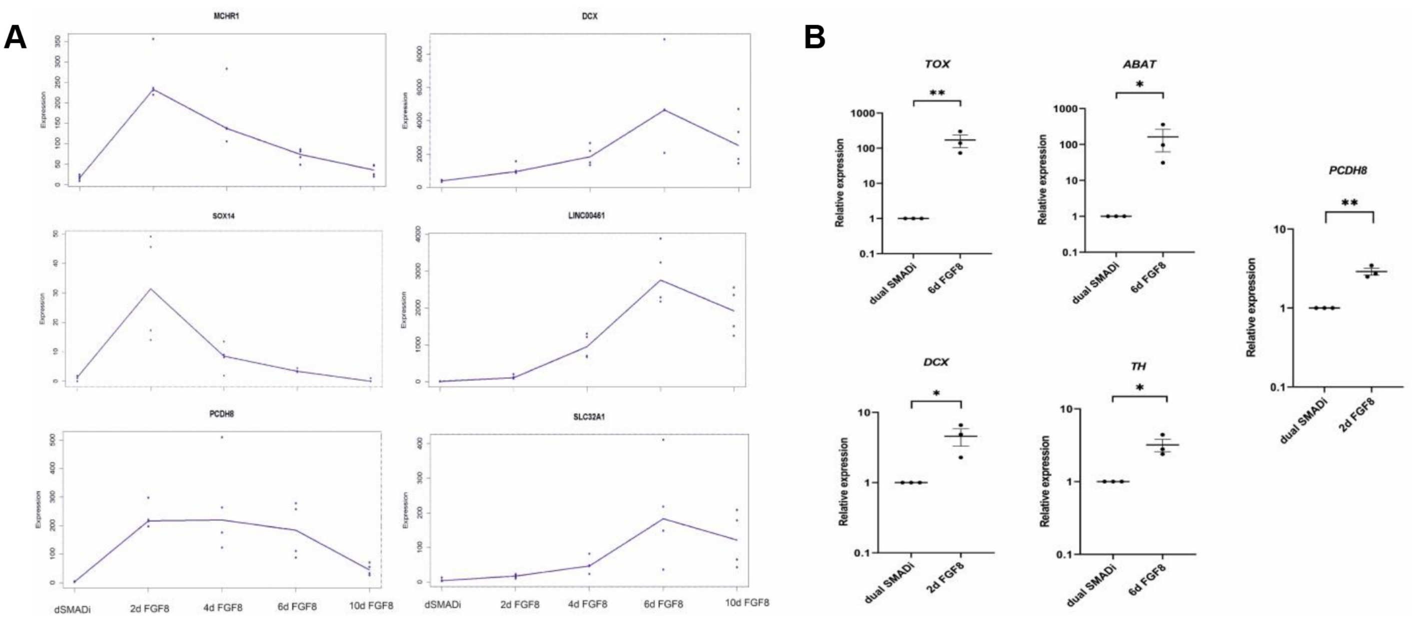

Fig. S7. Dynamic expression pattern of differentially expressed genes. (A) shows the dynamic expression paths Up-Down-Down-Down of *MCHR1, SOX14,* and *PCDH8.* and Up-Up-Up-Down of *DCX*, *LINC00461*, and *SLC32A1* across the four FGF8 treatment time points*.* The posterior probability (PP) for all six genes presented is more than 95% (PP>0.95). (B) *TOX*, *DCX, ABAT, TH,* and *PCDH8* were identified as genes which follow a dynamic expression path by EBSeqHMM analysis (PP≥0.95). All five of them displayed increase in their relative expression after 2d (d13) or 6d (d17) FGF8 treatment compared to dual-SMAD inhibition (dual SMADi). Dual SMADi, 2d FGF8 and 6d FGF8 samples were collected from three independent experiments (n=3). Statistical significance (ratio paired t-test) indicated as *(P≤0.05), ** (P≤0.01), and *** (P≤0.001). Related to Table S6.

Disease Models & Mechanisms • Supplementary information

Fig. S8.

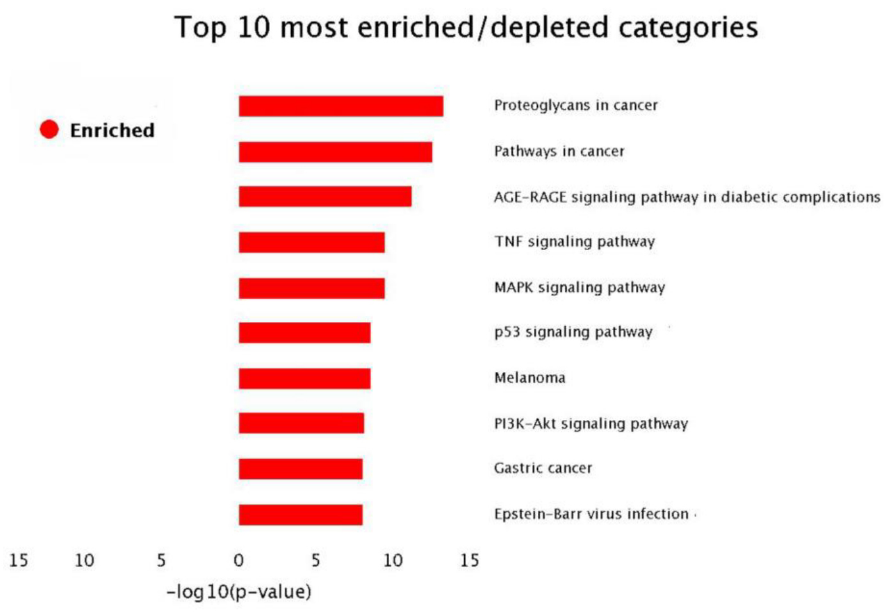

Fig. S8. Over-representation analysis (ORA). All the upregulated genes in the FGF8 and FGFR1 common mechanistic network were subjected to ORA and the top enriched (red) KEGG pathways (y-axis), and their respective p-values (x-axis) are presented here. Related to Fig. 7B.

Disease Models & Mechanisms • Supplementary information

Table S1. All the primers used in the study (related to Fig. 2, 4B, S2, S5 and S7)

Name	Sequence	Gene
qPCR_F	TGCCCAGTTTCCTCTTCAAT	*GNRH1*
qPCR_R	GTCAACTGGCAGAAACCCAA	*GNRH1*
qPCR_F	ACTCCCAGCCTGCTTCATAC	*PPIG/CycG*
qPCR_R	TACGTCTGAAACGATCCCTTG	*PPIG/CycG*
qPCR_F	TGTGTCGTGTTCTCAAAGGG	*MAP2*
qPCR_R	TGCATATGCGCTGATTCTTC	*MAP2*
qPCR_F	ATGGAGAAGCTGGTCATCCC	*LEFTY2*
qPCR_R	AGCTCTGGCTGAACCTCTTT	*LEFTY2*
qPCR_F	TGTATGAGAAGAACCAAACAGGT	*GAP43*
qPCR_R	CCACGGAAGCTAGCCTGAAT	*GAP43*
qPCR_F	GATTGCTCGGAAGTTGGTCT	*SPRY2*
qPCR_R	GGTCACTCCAGCAGGCTTAG	*SPRY2*
qPCR_F	CAGTCAACTTCAAGAGGCTTGG	*ETV5*
qPCR_R	TGCTCATGGCTACAAGACGAC	*ETV5*
qPCR_F	ACAGCTCAGCTCCACAGCAT	*FGFR3*
qPCR_R	GAGTCCTTGGGGACGGAG	*FGFR3*
qPCR_F	TCCCCCAACTACTCCAACGAC	*ASCL1*
qPCR_R	CCCTCCCAACGCCACTG	*ASCL1*
qPCR_F	TTGAGACGGTGGGGATGGTA	*DLX5*
qPCR_R	GGGGCATCTCCCCGTTTTT	*DLX5*
qPCR_F	CCGCACCCGTCAATGACTT	*FOXG1*
qPCR_R	CCGTCGTAAAACTTGGCAAAG	*FOXG1*
qPCR_F	GAGCGGCAGAAGTACCTGAG	*BARHL1*
qPCR_R	AGAAATAAGGCGACGGGAAC	*BARHL1*
qPCR_F	GAGAAGCCTTCAGAACTCCTTGC	*HES3*
qPCR_R	CTGCCGACCTCATCTCCGCG	*HES3*
qPCR_F	GTGATGCCAGATATACGAAACCC	*TOX*
qPCR_R	AGCTGTGACTGGTTAATGGTAGT	*TOX*
qPCR_F	GTTAGTCATGTGGGCATGTGTG	*DCX*
qPCR_R	TGGTGGAACCTCAGAGACTGAC	*DCX*
qPCR_F	CTTCCGTCTTCATCAGAGGC	*ABAT*
qPCR_R	CAGCTTCCAGCACAGCTACC	*ABAT*
qPCR_F	GGAAGGCCGTGCTAAACCT	*TH*
qPCR_R	GGATTTTGGCTTCAAACGTCTC	*TH*
qPCR_F	AAGACTTCCTTAGCCTTTCGG	*PCDH8*
qPCR_R	TGCTGTATCGGACTGTTTTGC	*PCDH8*

Disease Models & Mechanisms • Supplementary information

Table S2. All the antibodies used in the study (related to Fig. 3, S1a, S1b, S6a, S6b and S6c)

Primary antibodies				
Antigen	Supplier	Catalog	Species	Dilution
GNRH1	Gift from Erik Hrabovszky	EH#2000	Sheep	1:4000
GNRH1	Immunostar	20075	Rabbit	1:1000
FGFR1	Abcam	ab10646	Rabbit	1:100
FGFR1	Cell signaling technology	9740	Rabbit	1:200
NRP1	Abcam	ab81321	Rabbit	1:250
SPRY2	Santa Cruz	sc-100862	Mouse	1:150
GSX2	Merck	ABN162	Rabbit	1:500
ASCL1	Santa Cruz	sc-28688	Rabbit	1:100
FOXG1	Abcam	ab182659	Rabbit	1:1000
LHX2	Thermo Scientific	MA5-15834	Mouse	1:200
DLX5	Abcam	ab109737	Rabbit	1:500
DCX	Abcam	ab18723	Rabbit	1:200
Secondary antibodies				
anti-Mouse 488	Invitrogen	A32723	Goat	1:500
anti-Mouse 488	Invitrogen	A21202	Donkey	1:500
anti-Mouse 594	Invitrogen	A21203	Donkey	1:500
anti-Rabbit 488	Invitrogen	A21206	Donkey	1:500
anti-Rabbit 594	Invitrogen	A21207	Donkey	1:500
anti-Sheep 488	Invitrogen	A11015	Donkey	1:500

Table S3. Indicates the 18 activated downstream targets of FGF8 among the differentially expressed genes after two days of FGF8 (d13) detected by IPA.

Click here to download Table S3

Table S4. Contains the list of 1595 differentially expressed genes which are common during FGF8 treatment (two, four, six, and ten days). The table includes gene expression data (Log2FoldChange) at each of the four FGF8 treatment time points. Related to Fig. S4

Click here to download Table S4

Table S5. Shows the 84 differentially expressed genes which have a dynamic expression path upon FGF8 treatment and with a posterior probability (PP) of over 95% (PP>0.95).

Click here to download Table S5

Table S6. Shows the 24 differentially expressed genes out of 84 from Table S5, exhibiting a dynamic upregulation upon FGF8 treatment as indicated by their expression paths up-down-down-down, up-up-down-down or up-up-up-down. All 24 genes have a posterior probability (PP) of over 95% (PP>0.95). Related to Fig. S7.

Click here to download Table S6

Table S7. Contains the list of 266 differentially expressed genes which are common during FGF8 treatment (two, four, six, and ten days), and all of which belong to the mechanistic networks of FGF8 as well as FGFR1. The table includes the gene expression data (Log2FoldChange) at each of the four FGF8 treatment time points. Related to Fig. 7B.

Click here to download Table S7

Table S8. Contains the list of 190 differentially expressed genes (DEGs) out of the 266 DEGs in Table S7, showing differential expression also in Tdtomato enriched human GnRH neurons (Lund *et al.*, 2020). The table includes the gene expression data (Log2FoldChange) at each of the four FGF8 treatment time points and in Tdtomato enriched GnRH neurons (Lund *et al.*, 2020).

Click here to download Table S8
